# Recent trajectories of biometeorology in the Global South: a systematic review of biometeorology research in the International Journal of Biometeorology

**DOI:** 10.1007/s00484-026-03213-5

**Published:** 2026-05-07

**Authors:** Ariel S. Prinsloo, Claire Gallacher, Felix F. Adebayo, Betty Adegebo, Adnan Arshad, Shreya Banerjee, Elizabeth Carr, Thea J. Earnest, Cassia Holtz, Carmen Kganane, Alexi M. Marinaki, Chanice Mavudzi, L. Palesa Molefe, Ogone Motlogeloa, Nkosi Muse, Amanda Ndiweni, Leonardo B. Prado, Pippa J. Pryor, Sarah J. Roffe, Mahdieh Saed, Raghid Shehayeb, Adriaan J. van der Walt, Mukhtaar Waja, Michael J. Allen, Jennifer M. Fitchett, Peter J. Crank

**Affiliations:** 1 School of Geography, Archaeology and Environmental Studies, University of the Witwatersrand, Johannesburg, South Africa; 2 Leibniz Institute of Ecological Urban and Regional Development, Dresden, Germany; 3 Department of Geography and Environmental Management, University of Waterloo, Waterloo, Canada; 4 University of Lagos, Lagos, Nigeria; 5 State Key Laboratory of Grasslands & Agroecosystem, College of Pastoral Agriculture Science and Technology, Lanzhou University, Lanzhou, China; 6 Center for Emerging Technologies for Sustainable Development, Indian Institute of Technology Jodhpur, Jodhpur, India; 7 Department of Demography and Population Studies, School of Social Sciences, University of the Witwatersrand, Johannesburg, South Africa; 8 Center for the Environment, Harvard University, Cambridge, MA USA; 9 School of Geographical Sciences and Urban Planning, Arizona State University, Tempe, AZ USA; 10 School of Agriculture and Food Sustainability, The University of Queensland, Gatton, Australia; 11 Agrometeorology Division, Agriculture Research Council - Natural Resources and Engineering, Pretoria, South Africa; 12 Department of Geography, University of the Free State, Bloemfontein, South Africa; 13 Department of Geography & Environmental Planning, Towson University, Towson, MD USA

**Keywords:** PRISMA-style, Empirical, Global South, Publication trends, Equitable research

## Abstract

Literature reviews have become increasingly prominent in scientific discourse; however, few have critically examined the geographic distribution of research, particularly in the field of biometeorology, where studies remain disproportionately concentrated in the Global North. Building on previous regional reviews and special issues, this paper critically examines geographic disparities in publication trends, authorship, and methodological approaches to aid in identifying challenges and opportunities for equitable and justified future research. Using a PRISMA-style framework, 591 empirical studies conducted across 147 Global South countries were identified from a total of 3,046 publications in the International Journal of Biometeorology (IJBM) between 2000 and 2024. The review reveals a steady increase in contributions from the Global South over time, but highlights persistent gaps in representation, especially in author affiliation and research leadership. Thematic and methodological analyses highlight the diversity and innovation present in Global South contexts, while also revealing structural barriers to equitable participation in the field. This work advocates for more inclusive research practices and enhanced institutional support to bridge the divide between the Global North and South, fostering a more representative and collaborative future for biometeorology.

**Supplementary Information:**

The online version contains supplementary material available at 10.1007/s00484-026-03213-5.

## Introduction

Literature reviews have become increasingly ubiquitous across disciplines in the past decade (Kraus et al. 2022). Yet, even before this recent trend, there have been several biometeorological literature reviews (Beggs et al. 2017; Sheridan and Allen 2017; Fitchett 2021; Motlogeloa and Fitchett 2023) that have directly shaped the field and influenced the trajectory of the International Journal of Biometeorology (IJBM) toward more explicitly geographic scopes within the journal (Lecha Estela 2018; Dai et al. 2019; Fitchett 2025). Trends in specific biometeorological fields - such as Aeriobiology (Beggs et al. 2017), Climate and Human Health (Motlogeloa and Fitchett 2023), and Phenology (Donnelly and Yu 2017), demonstrate the increasing detail and expansion of the field. Geographic special issues in Latin America and the Caribbean (Lecha Estela 2018), Asia (Dai et al. 2019), and Africa (Fitchett 2021) have helped shift attention toward more Global South research, however biometeorology research dominance from the Global North remains (Sheridan and Allen 2017; Crank et al. 2025).

The disparities in biometeorological research output and authorship between the Global North and Global South have only recently come under direct scrutiny (Crank et al. 2025). Yet, when considering the geographically focused special issues of the past decade in the IJBM (Lecha Estela 2018; Dai et al. 2019; Fitchett 2025), the research being conducted within the geographic scope demonstrates strong scientific work within these broad geographic groupings, and persistent gaps even within these regions remain. In the Asia special issue of the journal, Dai et al. (2019) included 14 empirical studies conducted in Asia; however, upon further exploration, all but one of the studies (Nyssanbayeva et al. 2019 conducted research in Kazakhstan), were conducted in East Asian countries (China and South Korea), which are traditionally excluded from the Global South (Mahler 2017; Crank et al. 2025). A similar pattern appears within the Latin American and Caribbean special issue (Lecha Estela 2018), where not all featured papers are studies on these regions. Further potential exacerbations of this problem can stem from “extractive” research, a term used widely in justice literature (Gaudry 2011) or “parachuting” research, referring to instances where researchers from higher-income countries conduct fieldwork in lower-income regions, completing analysis and publication in their home institutions, with minimal ongoing engagement or collaboration with local scientists (Stefanoudis et al. 2021). Such approaches risk limiting knowledge exchange and capacity-building within the host country, reinforcing structural inequities in global research.

The special issue on Africa (Fitchett 2025) is the most recent issue to be completed following the identification of limited coverage of biometeorological research on the African continent as published in the IJBM (Fitchett 2021). While the trend of empirical studies is to be more representative of the Global South in this special issue, the concern around gaps in biometeorological research between the Global North and Global South is specifically highlighted (Crank et al. 2025). Collectively, these regional special issues highlight the ongoing desire and need to close gaps and inequities in biometeorological research between various countries and geographic regions.

To that end, Crank et al. (2025) highlighted the need for a more systematic consideration of the disparity in biometeorological research between Global North and Global South countries, regions, and researchers. Expanding upon the work of Crank et al. (2025), this study aims to examine the gaps in biometeorological knowledge in the Global South and demonstrate and determine trajectories of where efforts can be best aligned to close these gaps, reducing the existing (and widening) divide between the Global North and South, two broad (and terminologically unwieldy) categories. To do this, the paper seeks to identify biometeorological research (as published within the IJBM) that fits within a Global South delineation, determine research outputs for a proportion of time, and determine the implications of disparities between geographic regions in terms of representativeness and lingering obstacles. As a result, this review will aim to offer actionable insights to guide future research directions, editorial priorities and more equitable knowledge production in biometeorology.

## Methods

**Fig. 1:**
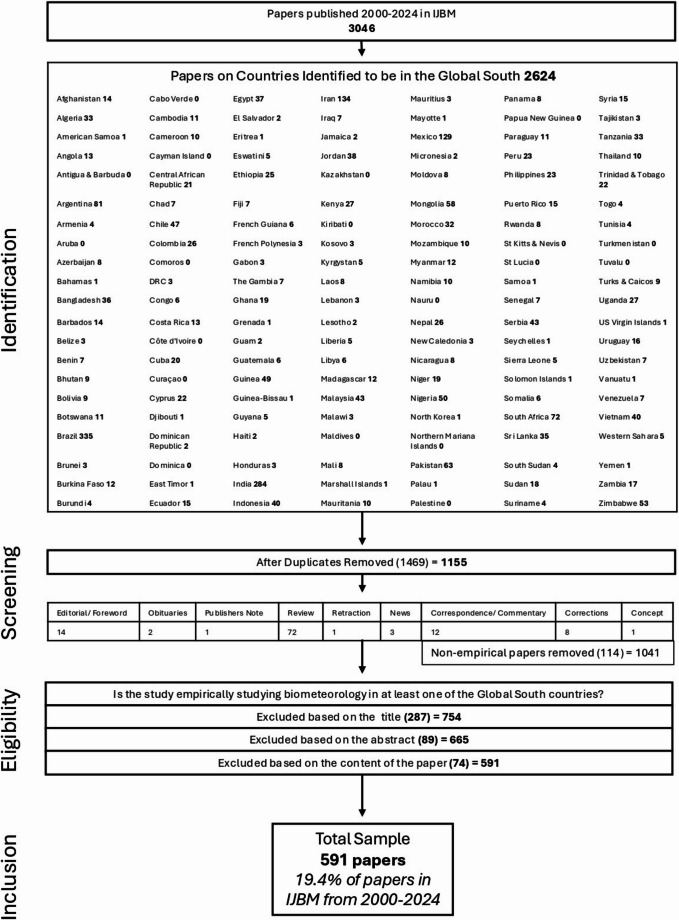
PRISMA-style diagram illustrating the process of article identification, screening, eligibility, and inclusion in this review

PRISMA, more generally used in the medical field, is a method of systematic review to report transparently on the reason, process and results of a review process (Page et al. 2021). The interdisciplinary field of biometeorology is broad and less medically based and therefore uses a PRISMA-style approach. This study is also informed by Fitchett (2021), who reviewed biometeorological research conducted on the African continent in a similar style, and published in the IJBM, to allow for effective cross-study comparison. Although biometeorological research is inherently interdisciplinary and consequently published across a wide range of journals, consistent with the prior reviews tracking engagement from the society and within the IJBM, this review considers only papers within this journal (Beggs et al. 2017; Donnelly and Yu 2017; Hondula et al. 2017; Sheridan and Allen 2017; Motlogeloa and Fitchett 2023). This approach allows for a coherent, internally consistent assessment of how the biometeorology community has engaged with Global South contexts. It also allows a targeted reflection on research trends, gaps and priorities within a leading journal in the field of biometeorology. Therefore, this study considers all papers published in IJBM, with volume and page numbers assigned, between the period 2000–2024. Through this period, a total of 3,046 papers were published in the IJBM (Fig. 1).

Paper identification and the downloading of all articles were conducted in July of 2025 at an in-person workshop run by the International Society of Biometeorology (ISB) Students and New Professionals (SNP), ensuring the finalisation of the final issue of 2024. The first step of a PRISMA-style approach to a systematic review involves the identification of relevant papers. In the case of a continent, such as Africa (Fitchett 2021), this is reasonably simple through the identification of all titles and text within the journal that contain the word ‘Africa’ and/or the current and former names of any of the 54 countries on the continent. When considering the Global South, the identification stage requires far more critical engagement to determine which countries should be included in the search functions required for that stage (Khan et al. 2022).

There is no universally agreed-upon list of countries that are considered to be part of the Global South, nor is there consistency in the way that this term is used (Mahler 2017; Crank et al. 2025). The purpose of this paper is not to put forward a singular definition of the Global South, nor claim authority on the countries that comprise this socio-economic grouping. However, in the interests of being able to perform a well-documented and consistent identification process for a PRISMA-style review, it was necessary to determine an operational list. This was developed through a three-stage process of consensus-building among the 26 authors, who stem from varied regions of the Global South and North (see supplementary file for a positionality statement). The first stage entailed the authors working in groups to identify on a map the broad regions, and countries therein, that they would consider to be part of the Global South. Bringing together these maps, consensus was quickly reached for several countries, as indicated through a voting process of all author-members. Where there was less consensus, a brief debate was entered into, followed by a vote. From this voting process, a list of countries likely to be in the Global South was developed. Thereafter, authors were tasked with identifying a reasonable reference (see supplementary file) that supports the classification of a list of countries allocated to them as being part of the Global South. This revealed many further sources that contain their own extensive lists of countries in the Global South. The decision was taken to maintain the vote-based list, and use supporting references for the purposes of excluding countries not supported by any literature to be included in the Global South, rather than to expand the list of countries. The process resulted in a final list of 147 countries that was agreed on by the authors through this process (Fig. 1).

Having identified the list of countries for inclusion in the study, the SpringerLink search function embedded in the IJBM website was used to identify and download papers from each of these 147 countries. This initial search yielded a total of 2,624 papers over the period 2000–2024 (Fig. 1). As a number of papers studied more than one country of interest, there were 1,469 duplicate papers which were thereafter removed in the screening process (Fig. 1). These duplicates were identified through uploading all of the papers into Zotero, and using the duplicate removal function.

The second stage of screening included the identification and removal of non-empirical papers, including editorials and forewords, obituaries, publishers’ notes, review papers, retractions (including removing the retraction note), news items, correspondence and commentaries, corrections, and conceptual papers which included no original data (Fig. 1). This resulted in the removal of a total of 114 papers, the majority of which (72) were review papers (Fig. 1).

Following this two-stage screening process, the remaining papers were evaluated for their eligibility by determining whether they conducted research *in* or *on* countries in the Global South. Inclusion criteria identified whether the names of countries merely appeared in discursive sections such as the introduction or discussion, without actually studying them. This was conducted through determining the country of study through the titles, abstracts and full texts. From this, a further 450 papers were excluded (Fig. 1). This yielded a total of 591 papers (see supplementary file), representing 19.4% of all publications in the IJBM in the period 2000–2024 (Fig. 1).

Once the papers for inclusion in the study had been finalised, each of the 591 papers were read with the purpose of understanding the content of the research, and extracting data on the countries in the Global South studied, the publication year, number of authors, the countries of the authors’ affiliations, the subfield of biometeorology, the data types, and the methods of analysis. These were used in compiling figures to demonstrate the diversity in biometeorological research in the Global South, and track publication trends through space and time. Thematic engagement with the content of the papers informed the broader areas of debate and critical engagement, and how this has evolved through time.

## Results and Discussion

### Publication trends

**Fig. 2:**
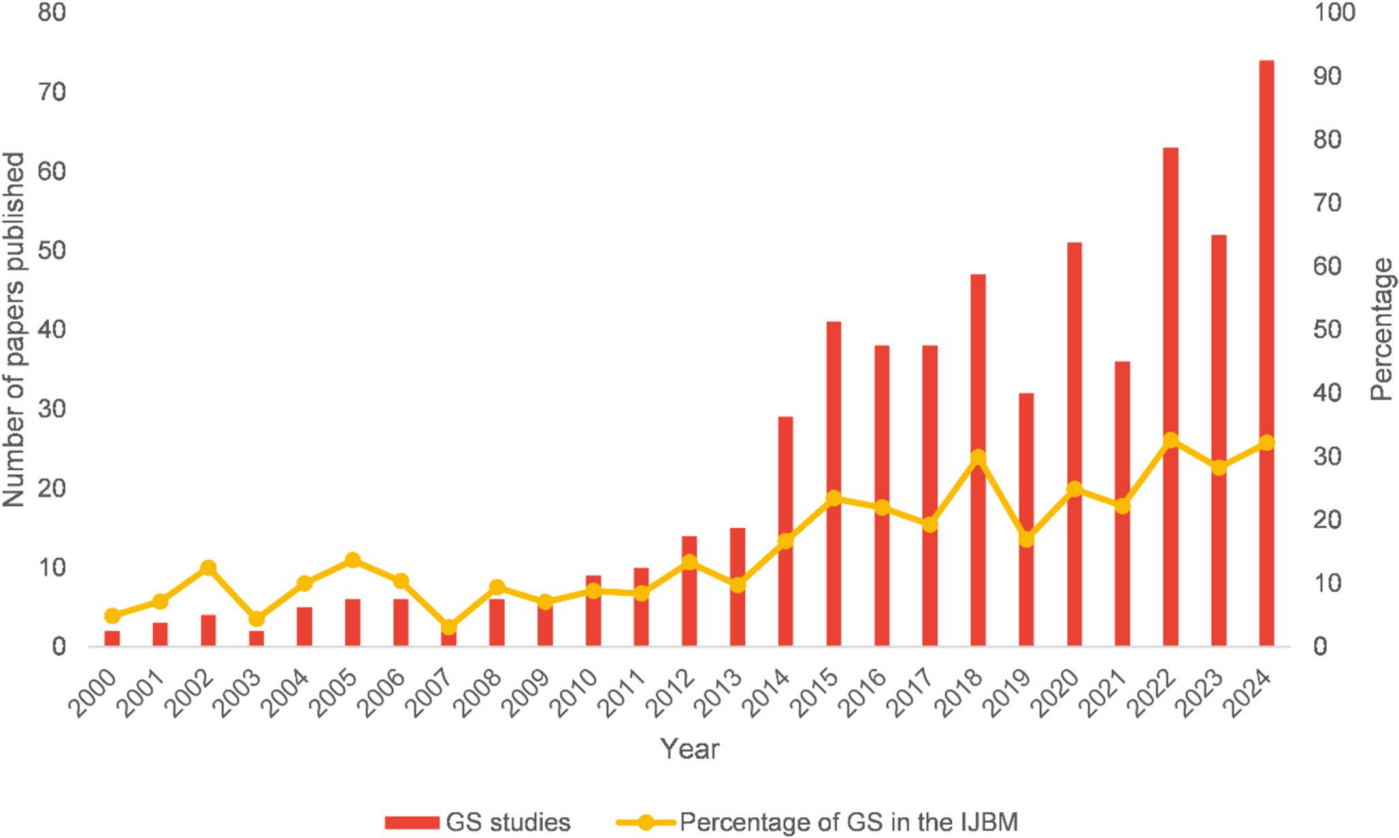
Number of papers published per year from the Global South and percentage of Global South studies in the IJBM, between 2000–2024

Between 2000 and 2024, there has been a clear upward trajectory in the number of biometeorological studies published in the Global South (Fig. 2). During the early years of this period, annual outputs remained relatively low, seldom surpassing ten publications per year (Fig. 2). This limited productivity may reflect infrastructural constraints, restricted access to funding, or lower visibility and identification of fields that fit within biometeorological research within these regions. From 2010 onward, a steady increase in publication volume is evident, with a marked increase observed after 2014 (Fig. 2). This culminated in a peak of 74 publications in 2024, reflecting growing engagement with biometeorological themes across diverse Global South contexts. The expansion in scholarly output may correspond with heightened awareness of climate-related challenges, rapid urbanisation, and public health concerns specific to these regions. Further, the ISB has endeavoured to increase engagement of scholars through their congresses held every three years as well as workshops held by ISB regional councillors to increase awareness of the IJBM. This is supported by Keatley (2017) and Sheridan and Allen (2017) and mirrors trends in academic publications in general (Gu and Blackmore 2016).

**Fig. 3:**
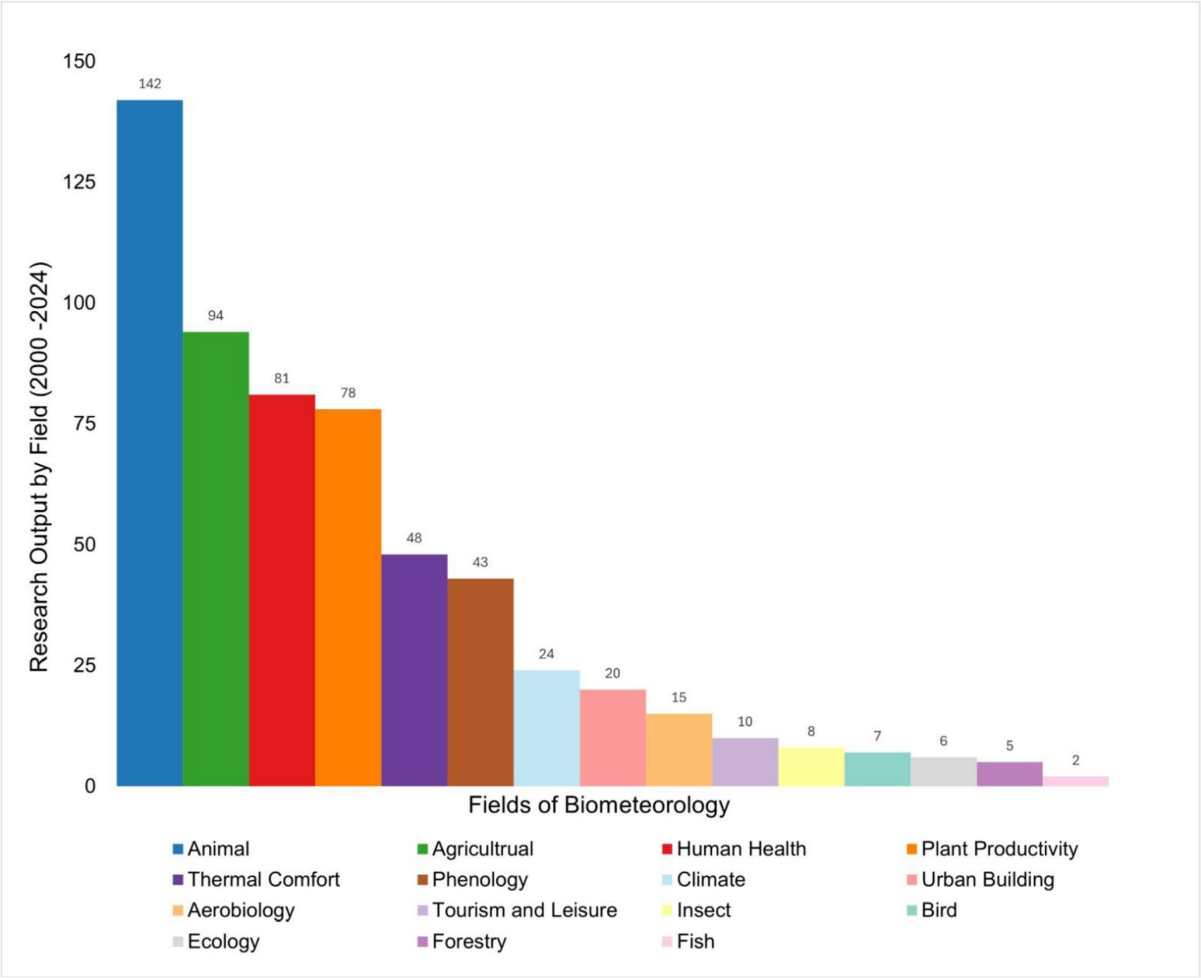
Biometeorological research areas of papers published in the Global South

A diverse array of subfields within biometeorology can be identified from research conducted in the Global South (Fig. 3), though some thematic areas remain difficult to delineate due to interdisciplinary overlaps such as thermal comfort. Consistent with findings from the African biometeorological review (Fitchett 2021), animal biometeorology constitutes the most represented category with 142 publications (Fig. 3). Agricultural biometeorology follows with 93 publications while research focused on ichthyological (fish-related) biometeorology remains comparatively limited in Global South literature (Fig. 3). Comparable in publication output were the fields of human health, plant productivity, thermal comfort, phenology, climate and urban building (Fig. 3). Animal and agricultural biometeorology are perhaps more published as they directly research food security as national economic priorities - and may therefore attract more funding. For more niche disciplines, there may be disciplinary spread into subject-related journals.

### Authorship

**Fig. 4:**
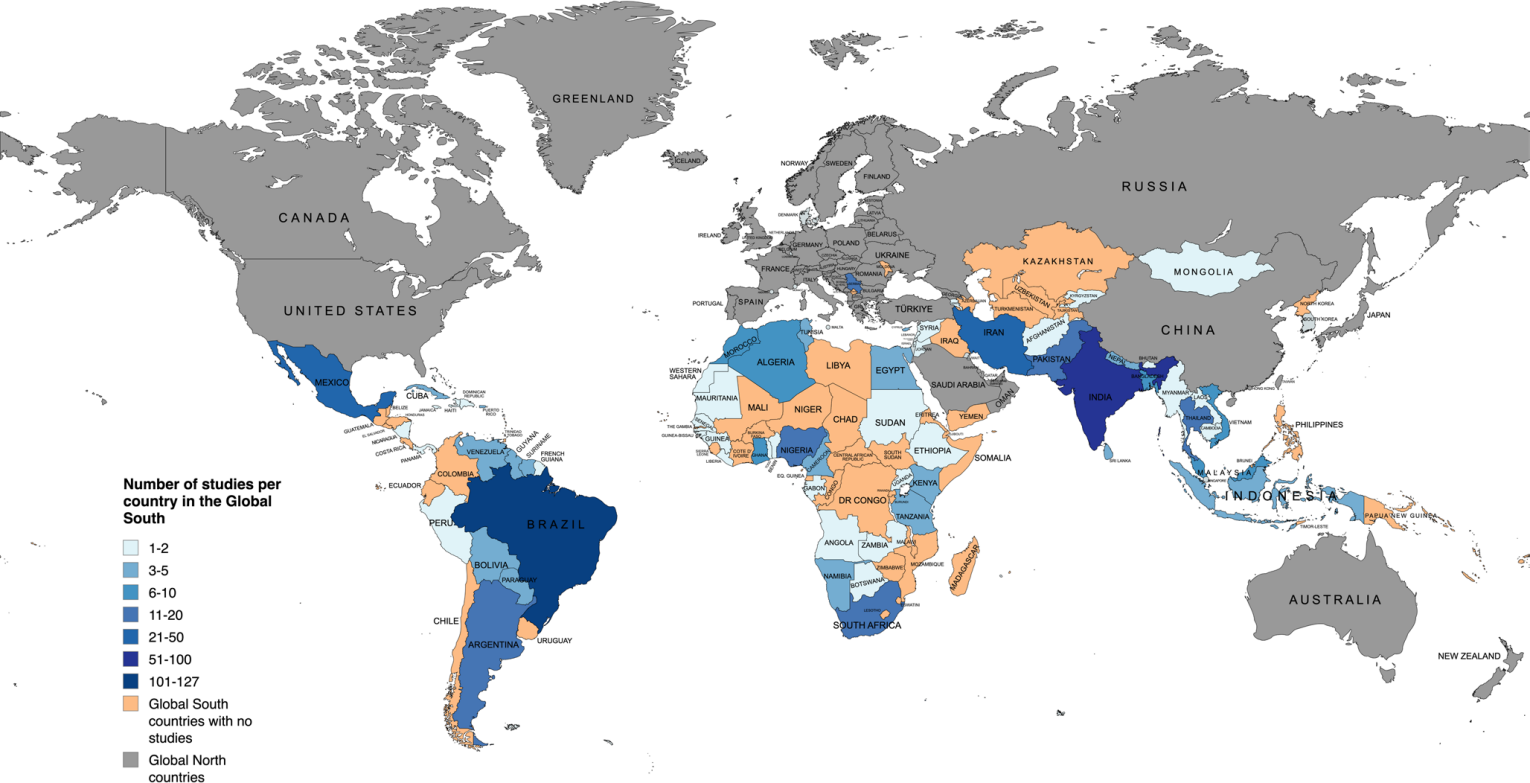
Number of studies per country in the Global South

The spatial distribution of biometeorological studies across the Global South reveals pronounced disparities in research output, with a concentration of publications originating from a limited subset of countries (Fig. 4). Brazil emerges as the leading contributor with 127 publications, followed by India (97 publications), Iran (48 publications), Mexico (25 publications), Nigeria (18 publications), and South Africa and Argentina (17 publications respectively), each demonstrating relatively high levels of research activity (Fig. 4). In contrast, substantial regions - including much of Africa, Central America, and Central and Southeast Asia - are represented by only a handful of publications, often limited to one or two per country (Fig. 4). These patterns reflect an uneven landscape of scholarly production, wherein research agendas and authorship are disproportionately shaped by countries with greater institutional capacity and access to resources. This imbalance raises concerns regarding the representativeness of biometeorological scholarship in the Global South, as the underrepresentation of certain regions may obscure localised challenges and limit the applicability of broader research findings across diverse contexts. Further, this raises concerns regarding global models that rely heavily on data from the Global North, as such imbalances can bias model outputs toward Global North conditions, undermining their representativeness and necessitating careful application in global contexts.

**Fig. 5:**
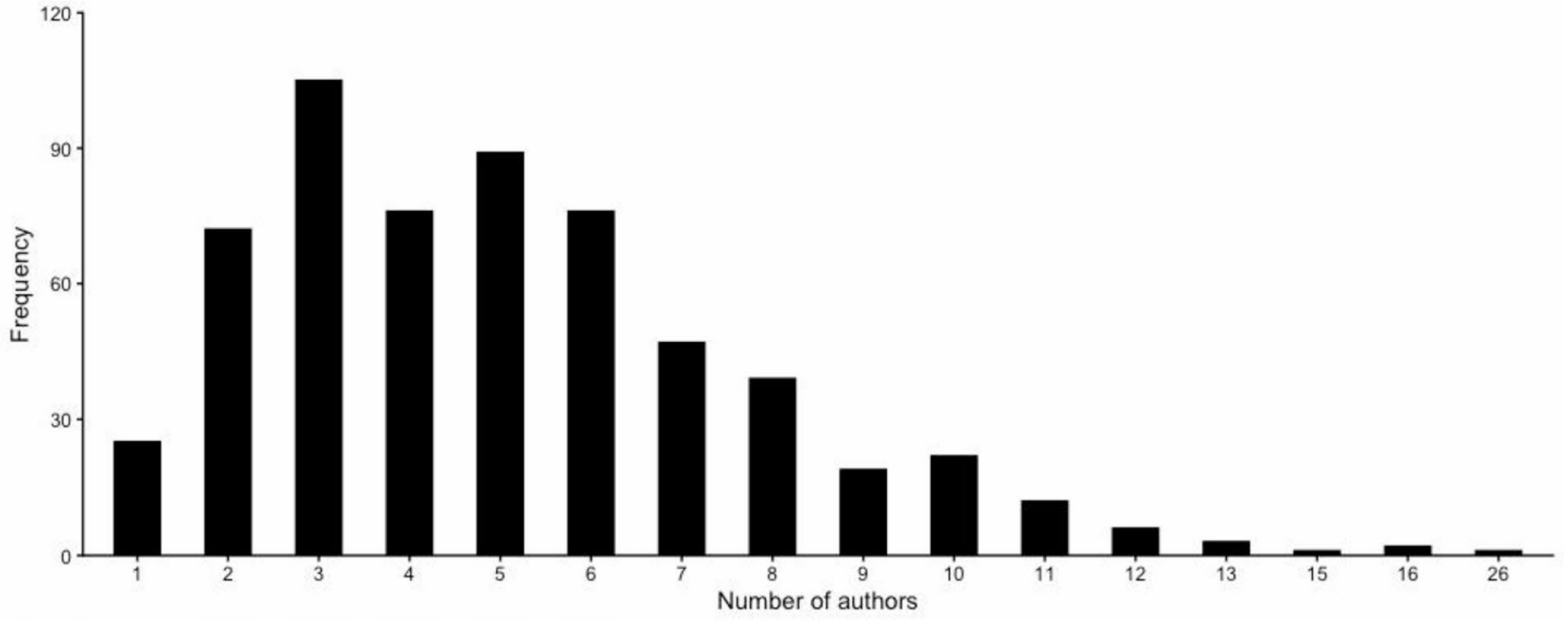
Number of authors per article

The number of authors per article varies considerably across the reviewed publications (Fig. 5). The most common author counts were for three and five authors, reflecting moderate-sized research teams (Fig. 5). However, several papers featured substantially larger collaborative groups, with one study including as many as 26 authors (Fig. 5). This variation in authorship suggests a high degree of collaborative engagement within biometeorological research conducted in the Global South. Such collaboration may be indicative of interdisciplinary approaches, multi-institutional partnerships, and the growing complexity of research questions addressed in these contexts.

**Fig. 6:**
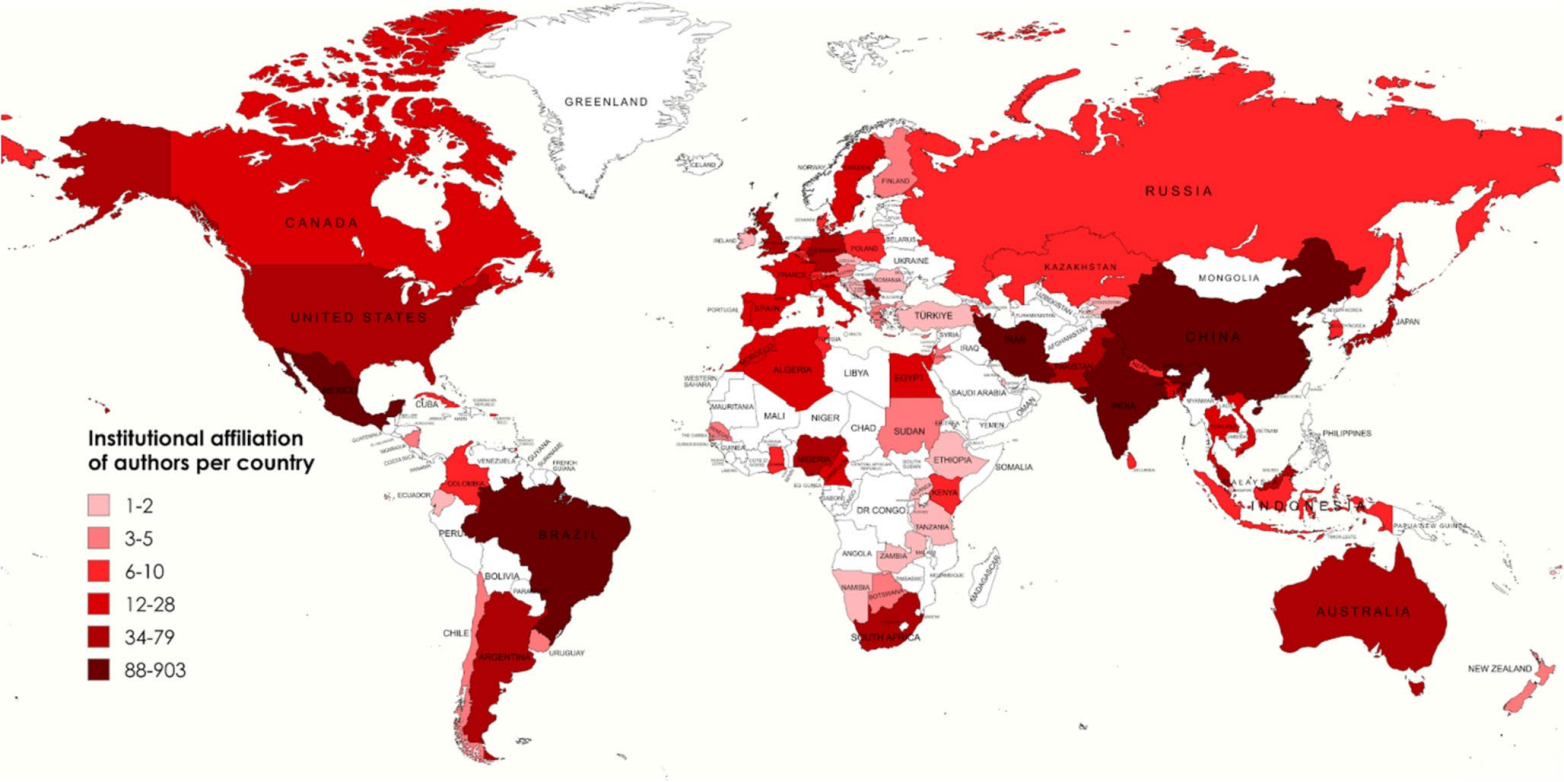
Institutional affiliation of authors for studies conducted in the Global South

Institutional affiliation patterns were examined to better understand the geographic and institutional landscape of authors contributing to biometeorological research in the Global South (Fig. 6). This analysis provides insight into patterns of research funding, resource allocation, and the visibility of institutions within the field. A concentration of country-specific author affiliations in countries such as Brazil (903), India (634) Iran (219) and Mexico (112) indicate strong domestic research capacity and institutional engagement within and across these individual regions (Fig. 6). The most common affiliations in Africa are South Africa (66) followed by Nigeria (43) (Fig. 6). Within South America, after Brazil, the next highest affiliation is Argentina (72) followed by Chile (5) (Fig. 6). In addition, a notable proportion of authors are affiliated with institutions in the Global North, including the United States of America (79), Australia (74), Germany (74), the United Kingdom (34), Canada (16), France (12) and Russia (8), despite the studies being conducted in Global South contexts (Fig. 6). This may reflect temporary institutional placements of researchers originally from the Global South, institutional affiliations to both a Global North and South context, collaborative international partnerships, funding partnerships or instances of “parachute science”, wherein researchers from the Global North conduct studies in the Global South without sustained local engagement (Serwadda et al. 2018; Stefanoudis et al. 2021). These patterns underscore the complex dynamics of research collaboration and highlight the importance of critically examining authorship and affiliation to ensure equitable and justified representation and capacity-building within Global South biometeorological scholarship.

### Methodological insights from the Global South

**Fig. 7:**
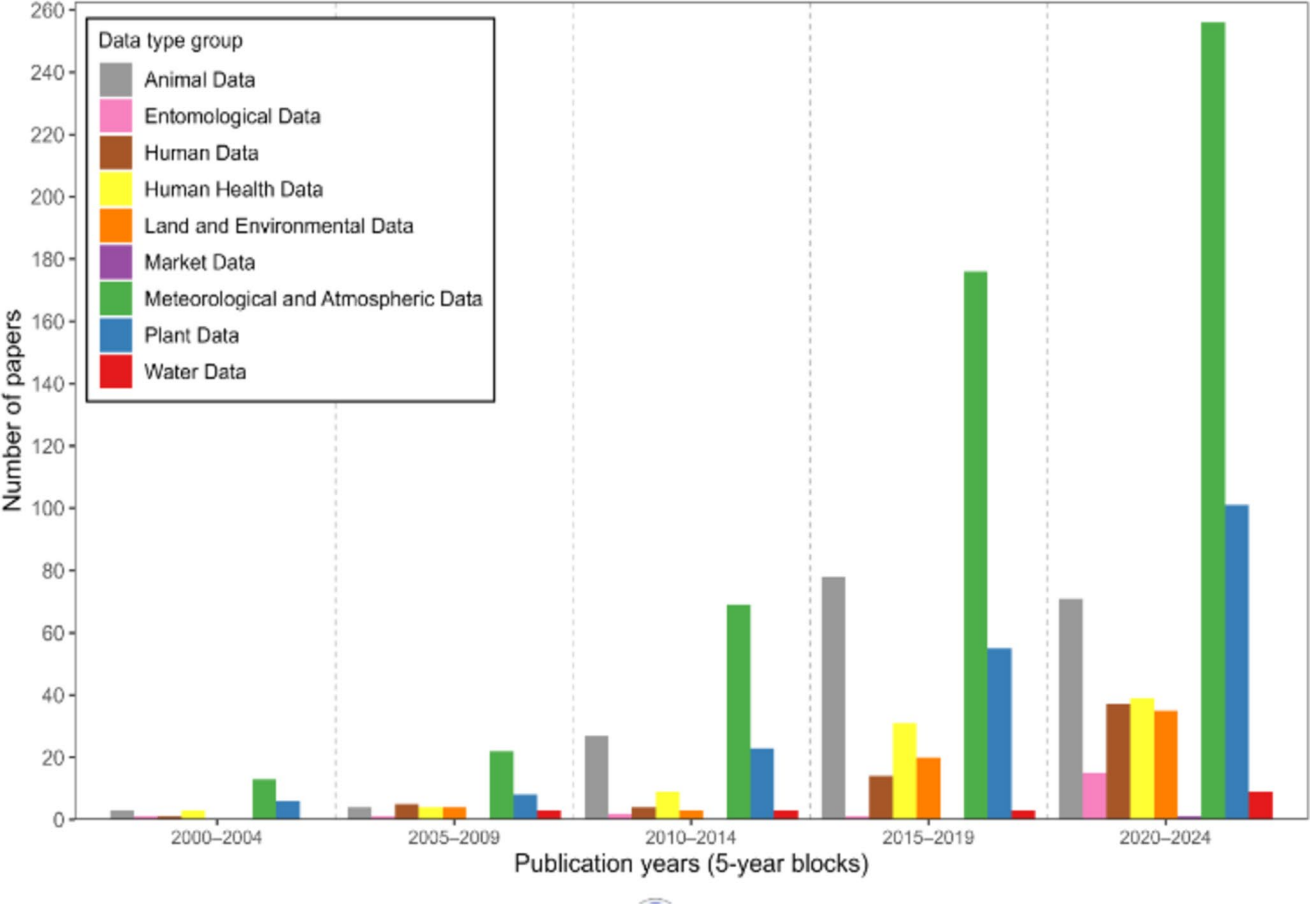
Types of data informing Global South research between 2000–2024

The temporal distribution of publications utilizing different data types demonstrates a pronounced shift over the review period (Fig. 7). Contributions have shifted from more ‘traditional’ methodologies and introduced new methodological and thematic directions, including applications of neural networks, artificial intelligence, model development and geospatial technologies such as GIS and remote sensing, indicating a growing diversification and technological advancement in Global South biometeorological research. When grouped into five-year intervals, early phases (i.e., 2002–2004 and 2005–2009) exhibit minimal activity, with most categories such as animal data, human health data, and water-related data, remaining below 20 publications (Fig. 7). This is consistent with fewer publications in this period where the increase from 2010-2014 period similarly coincides with the increase in publications. A notable increase in the types of data used occurs after 2010, culminating in a substantial surge during 2020–2024 (Fig. 7). In this most recent interval of 2020–2024, meteorological and atmospheric data dominates (~ 250 publications), followed by plant data (~ 100 publications) and animal data (~ 70 publications) (Fig. 7). These patterns underscore a growing emphasis on environmental and biological datasets in Global South biometeorological research, reflecting both the diversification of data sources and the increasing methodological sophistication of the field. This could speak to the growing capacity of specific researchers in the Global South to obtain datasets (Serwadda et al. 2018) and the accessibility of large global datasets, that are presumed to be accurate but require ground truthing in many contexts (Mehmood 2021; West et al. 2023; Láng-Ritter et al. 2025).

**Fig. 8:**
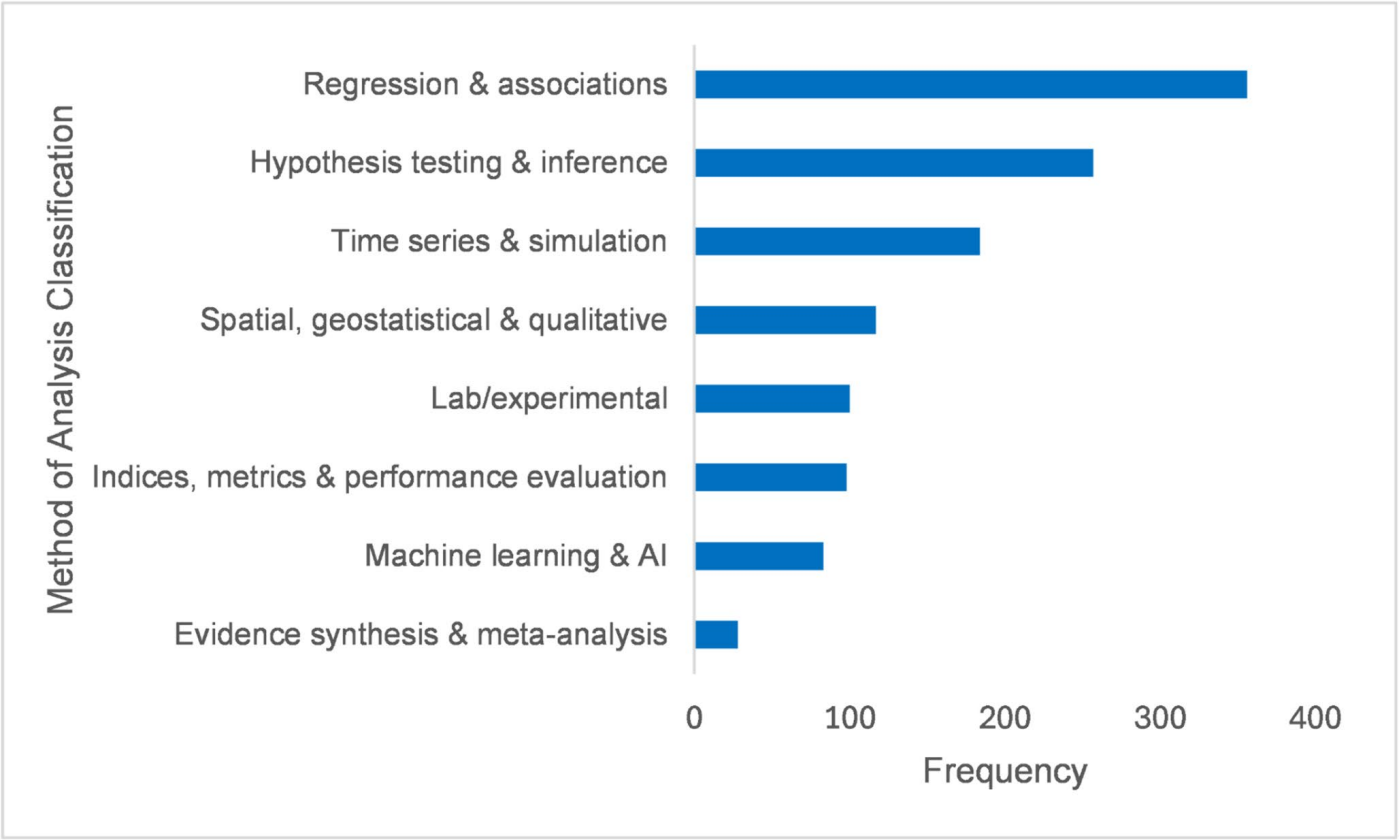
Methods of data analysis used in Global South research papers

Methodological approaches in Global South biometeorological research span diverse analytical frameworks (Fig. 8). Regression-based analyses and association testing dominate, with a frequency of 356 instances identified in the publications (Fig. 8), reflecting a continued reliance on statistical approaches to explore climate-health linkages, environmental drivers, and biological responses. Hypothesis testing and inferential statistics form the next major group with 257 instances identified, underscoring the field’s grounding in classical statistical approaches. Time-series analyses and simulation models feature prominently with 184 instances, indicating growing engagement with temporal dynamics, forecasting, and scenario-based assessments. Spatial, geostatistical, and qualitative methods are also common with 117 instances, highlighting the importance of spatial heterogeneity, microclimates, and contextualised environmental observations in Global South settings. By contrast, laboratory and experimental studies remain rare with 100 instances identified, likely due to structural constraints such as limited access to specialised facilities and long-term infrastructure. These gaps are compounded by the prevalence of research led or co-led by Global North institutions. Present approaches such as machine learning and artificial intelligence, are increasingly evident with 83 instances, reflecting improved computational capacity and access to open-source tools, though their adoption is concentrated in countries with stronger research infrastructure. Evidence synthesis and meta-analysis appear in only a handful of studies, with 28 instances, suggesting that systematic aggregation of findings remains underdeveloped, constrained by the scarcity of long-term or multi-site datasets. Collectively, these patterns reveal a field anchored in traditional statistical methods but gradually diversifying through computational, geospatial, and model-based techniques. The limited presence of advanced experimental work underscores persistent inequities in infrastructure and leadership, reinforcing the need for targeted investment and equitable collaboration to advance methodological innovation across the Global South.

### Reflections on the excluded material

In any review paper, the criteria for inclusion and exclusion of publications is critical in framing the work under consideration. For this study, the first - and most difficult - decision related to which countries were considered part of the Global South. To ensure a robust process, this involved an iterative process, working with a group of over 20 biometeorologists. Despite the rigour in the approach, we do not argue that this represents a single, definitive list of countries in the Global South. By documenting these included countries, we allow reproducibility of these results, while recognizing that the decisions to include or exclude individual countries in compiling this list will inherently influence the output results. Beyond this, we excluded all review papers, to prevent a circular referencing of reviews within reviews. This does, however, mean that review papers emanating from and with a critical focus on the Global South would be excluded. Finally, we excluded papers that were uploaded to the IJBM early access list, but which did not yet have volume and page numbers. This meant that the first papers accepted towards the first African special issue on Biometeorology (Fitchett 2025) were not included.

### Encouraging greater participation in the International Society of Biometeorology

Enhancing the participation of Global South researchers in the ISB is essential for achieving equitable and justified global representation as well as meeting the initial aims set out by the society. Professional societies such as the ISB play a critical role in fostering networking, mentorship, and resource-sharing. Recent initiatives by the ISB, including the establishment of regional councillor positions for Africa, Latin America, and Asia, and targeted outreach through the SNP group, represent important steps toward inclusivity (Crank et al. 2024). Building on these efforts, further measures could strengthen Global South engagement. Financial support mechanisms, such as travel grants and reduced conference fees, would enable researchers from low-income and middle-income countries to attend international meetings and congresses. Hosting conferences, workshops, and training schools within Global South regions would reduce access barriers, amplify local priorities, and facilitate the development of regional research networks. Additionally, structured mentorship programs pairing early-career scientists from the Global South with experienced researchers internationally could promote skills transfer and foster more equitable collaborative opportunities.

## Conclusion

This review paper reveals meaningful progress in biometeorology across the Global South, alongside persistent structural inequalities in where research is conducted and who leads it. Publication outputs from the Global South have increased over the past two decades, yet they remain concentrated in a small number of countries, and many of the most climate-vulnerable regions continue to be underrepresented in biometeorological research. Local leadership is rising but still limited, with Global North institutions frequently driving research agendas. These imbalances restrict the capacity of the field to generate contextually relevant knowledge. Addressing these gaps will require deliberate commitments to equitable collaboration, improved access to data and research infrastructure, and expanded opportunities for Global South scientists to participate in global scientific networks. Professional societies such as the ISB play a key role by strengthening mentorship, supporting travel and training, and increasing the geographical diversity of conferences and workshops. A more geographically balanced biometeorology field is not only a matter of fairness; it is foundational to advancing contextual scientific understanding. Realising this goal ensures that communities across the Global South are equipped to confront the growing challenges of climate and weather extremes and that scientific knowledge and the benefits it yields are relevant and shared across geographies.

## Supplementary Information

Below is the link to the electronic supplementary material.

Supplementary Material 1 (PDF 900 KB)

## Data Availability

As a review paper, the data used are papers published in the IJBM. We have included a full list in the supplementary file.
