# Supplementary material for: Recent trajectories of biometeorology in the Global South: a systematic review of biometeorology research in the International Journal of Biometeorology
Source: Int J Biometeorol. 2026 May 7;70(5):156. doi: 10.1007/s00484-026-03213-5 (PMC13152919; doi:10.1007/s00484-026-03213-5)
Supplement: Supplementary file 1 — Supplementary Material 1 (PDF 900 KB) [file 484_2026_3213_MOESM1_ESM.pdf]

Supplementary File

Journal: International Journal of Biometeorology

Title: Recent trajectories of biometeorology in the Global South: A systematic review of biometeorology research in the International Journal of Biometeorology.

Ariel S. Prinsloo*[1], Claire Gallacher[2], Felix F. Adebayo[3], Betty Adegebo[4], Adnan Arshad[5], Shreya Banerjee[6], Elizabeth Carr[1], Thea J. Earnest[1], Cassia Holtz[1], Carmen Kganane[1], Alexi M. Marinaki[1], Chanice Mavudzi[1], L. Palesa Molefe[1], Ogone Motlogeloa[7], Nkosi Muse[8], Amanda Ndiweni[1], Leonardo B. Prado[9], Pippa J. Pryor[10], Sarah J. Roffe[11,12], Mahdieh Saed[3], Raghid Shehayeb[2], Adriaan J. van der Walt[12], Mukhtaar Waja[1], Michael J. Allen[13], Jennifer M. Fitchett[1], Peter J. Crank[3]

[1]School of Geography, Archaeology and Environmental Studies, University of the Witwatersrand, Johannesburg, South Africa

[2]Leibniz Institute of Ecological Urban and Regional Development, Dresden, Germany

[3]Department of Geography and Environmental Management, University of Waterloo, Waterloo, Canada

[4]Independent Researcher, Lagos, Nigeria

[5]State Key Laboratory of Grasslands & Agroecosystem, College of Pastoral Agriculture Science and Technology, Lanzhou University, Lanzhou, China

[6]Center for Emerging Technologies for Sustainable Development, Indian Institute of Technology Jodhpur, Jodhpur, India

[7]Department of Demography and Population Studies, School of Social Sciences, University of the Witwatersrand, Johannesburg, South Africa

[8]Center for the Environment, Harvard University, Cambridge, Massachusetts, United States of America

[9]School of Geographical Sciences and Urban Planning, Arizona State University, Tempe, Arizona, United States of America

[10]School of Agriculture and Food Sustainability, The University of Queensland, Gatton, Australia

[11]Agrometeorology Division, Agriculture Research Council - Natural Resources and Engineering, Pretoria, South Africa

[12]Department of Geography, University of the Free State, Bloemfontein, South Africa

[13]Department of Geography & Environmental Planning, Towson University, Towson, Maryland, United States of America

*corresponding author: Ariel.Prinsloo@wits.ac.za

1) Total number of papers following PRISMA-style review.

No	Publication Year	Author/s	Number of authors	Title	Url	Vol	Iss	Pages	GS country/ies studied (list all)	Region (ie continent)	Countries of authors' affiliations (list all)	Field of Biometeorology	Data type/s	Data analysis methods
1	2018	Rymbai, Dayo	4	The insight of	http://link.	62	10	1833-1845	India	Asia	India	Phenology Commi	Survey	Tobit model (Cens
2	2004	Oguntunde,	4	Crop growth a	http://link.	49	2	106-112	Ghana	Africa	Germany and Netherl	Phenology Commi	Measurements of	Surface alpha mo
3	2021	Nunfam, Vict	10	Estimating the	https://lin	65	12	2059-2075	Ghana	Africa	Australia, Ghana,	Climate and Huma	Questionnaires, t	Descriptive statist
4	2020	Nunfam, Vict	10	Barriers to occ	http://link.	64	7	1085-1101	Ghana	Africa	Australia, Ghana,	Climate and Huma	Focus group disc	Nvivo, IBM Statisti
5	2021	Ngo, Hieu K.	7	Impact of tem	https://lin	65	7	1205-1214	Vietnam	Asia	Vietnam, Australia	Climate and Huma	Daily hospital adr	regression analys
6	2023	Mbouna, Am	5	Malaria metric	https://lin	67	1	93-105	Camaroon	Africa	Cameroon, Ghana, U	Climate and Huma	rainfall and tempe	vector borne dise
7	2022	Masinaei, Ma	1	Estimating the	https://lin	66	6	1125-1144	Iran	Asia	Iran	Climate and Huma	Cumulative rainfa	Moran I, Bayesian
8	2021	Kwembeya, E	1	Tracking biolo	https://lin	65	4	577-586	Namibia	Africa	Namibia	Phenology Commi	Forty-nine herba	Microsoft Excel sp
9	2020	Hebbar, K B;	4	Effect of eleva	https://lin	64	1	47-57	India	Asia	India, US	Phenology Commi	Min Temperature	ANOVA, Open To
10	2017	Frimpong, Kw	1	Heat exposur	https://lin	61	3	397-406	Ghana	Africa	Autralia	Climate and Huma	Temperature, hu	WBGT
11	2018	Asamoah, Be	3	Is ambient hea	http://link.	62	3	319-330	Ghana	Africa	Sweden, Cyprus, Aus	Climate and Huma	Pregnancy woma	WBGT, Survey, Si
12	2018	Acharya, Bip	6	Modeling the	http://link.	62	11	1973-1986	Nepal	Asia	China, Germany, Nep	Climate and Huma	Dengue data;pop	Ordinary least squ
13	2002	Stuntz, Sabir	3	Rainforest air-	http://link.	46	2	53-59	Panama	Central America	Germany and Switzer	Phenology Commi	Temperature, ev	ANOVA
14	2006	Bendix, J.; Ho	7	Seasonality o	http://link.	50	6	370-384	Ecuador	South America	Germany	Phenology Commi	wind, soil/air temp	time series analys
15	2024	Gutiérrez, Ju	3	Causal assoc	https://lin	68	10	2003-2013	Colombia	Central America	Colombia	Climate and Huma	Epidemiological	Directed acyclic g
16	2022	Fitchett, Jenn	3	Advance in th	https://lin	66	11	2251-2258	South Africa	Africa	South Africa	Phenology Commi	Daily Max, Minimu	arithmetic averag
17	2023	Marques, Jor	7	Thermal exch	https://lin	67	7	1237-1249	Brazil	South America	Brazil	Animal Biometeor	air temperature, h	Temperature hum
18	2019	Li, Wantong;	9	The variation	http://link.	63	7	847-860	Cross countries	Africa	China, USA, United Ki	Phenology Commi	Global Land Surf	Pearson Correlati
19	2023	Kaissassou,	6	A 43-year of h	https://lin	67	12	2069-2080	Central Africa	Africa	Camaroon	Climate and Huma	UTCI through ER/	Spatial and Temp
20	2020	Hossain, Moh	6	Weather facto	http://link.	64	4	561-569	India	Asia	Australia, China, Balg	Climate and Huma	Data on pneumo	Cross-correlation
21	2024	Gonçalves, M	4	Optimizing ev	https://lin	68	1	57-67	Brazil	South America	Brazil	Phenology Commi	irrigation system	Crop evapotransp
22	2013	Eludoyin, Oye	5	The physiolog	http://link.	57	2	241-264	Nigeria	Africa	Nigeria	Climate and Huma	air temperature a	ANOVA, Effective
23	2008	Boonprong,	5	Productivity o	http://link.	52	5	409-415	Thailand	Asia	Thailand and German	Animal Biometeor	Temperature, hu	Temperature hum
24	2023	Ali, Asep I. M.	4	Physiological	https://lin	67	11	1757-1764	Indonesia	Asia	Indonesia	Animal Biometeor	Temperature, Hu	Temperature hum
25	2013	Akinbobola,	2	Predicting Ma	http://link.	57	5	721-728	Nigeria	Africa	Nigeria	Climate and Huma	rainfall, relative h	ARIMA
26	2023	Ake, Ayodele	5	Melatonin mo	https://lin	67	2	389-404	Nigeria	Africa	Nigeria	Animal Biometeor	Tweinty Healtly d	Temperature hum
27	2014	Yamada, Elia	2	Agro-climatic	http://link.	58	9	1995-2010	Brazil	South America	Brazil	Phenology Commi	Mean annual tem	Multiple linear reg
28	2016	Minka, Ndazo	3	Effect of wet-	http://link.	60	3	373-380	Nigeria	Africa	Nigeria	Animal Biometeor	Temperature, hu	Onboard thermal
29	2012	Minka, N. S.;	2	Assessment c	http://link.	56	2	333-341	Nigeria	Africa	Nigeria	Animal Biometeor	wet- and dry-bull	Student's t test, P
30	2016	Akbari, Mohs	2	Effects of add	http://link.	60	6	867-871	Iran	Asia	Iran	Bird	Meteorological, A	Two-way Analysis
31	2015	Zakari, Friday	4	Effects of age	http://link.	59	12	1813-1824	Nigeria	Africa	Nigeria	Animal	Blood sample, Me	GraphPad Prism,
32	2015	Sinkalu, Vict	4	Ameliorative e	http://link.	59	1	79-87	Nigeria	Africa	Nigeria	Bird	Meteorological (d	Two-way analysis
33	2018	Sinkalu, Vict	2	Combined eff	http://link.	62	1	09-15	Nigeria	Africa	Nigeria	bird	Meteorological, a	Statistical Packag
34	2014	Che Salmah,	5	Distribution of	http://link.	58	5	679-690	Malaysia	Asia	Malaysia	Insect	Physico-chemic	Multiple regressio
35	2022	Prabakar, Go	10	Seasonal vari	https://lin	66	8	1547-1560	India	Asia	India, USA	Bird	Meteorological (n	ANOVA (using SA
36	2022	Ramos Perei	4	How aerial ins	https://lin	66	3	601-612	Brazil	South America	Brazil, Portugal	Bird	Animal and Mete	Song Meter 2 (SM
37	2012	Omonijo, Aki	4	Effect of therm	https://lin	56	5	873-885	Nigeria	Africa	Nigeria, Germany	Human Health	Human Health, M	Stepwise regressi
38	2021	Olutumise, A	3	Effect of clima	https://lin	65	6	951-961	Nigeria	Africa	Nigeria, USA	Human Health	Structured Quest	Descriptive statist
39	2024	Obe, Oluwafe	3	An assessme	https://lin	68	5	811-828	Nigeria	Africa	Ireland, Hong Kong	Human Health	Meteorological D	Weather Researc
40	2024	Sharafi, Milac	4	Integration of	https://lin	68	2	237-251	Iran	Asia	Iran, Fiji	Plant productivity	Meteorological D	Support Vector R
41	2019	Ramos, Rodr	6	Climate mode	http://link.	63	3	281-291	Brazil	South America	Brazil, Australia	Plant productivity	Meteorological a	CLIMEX Modeling
42	2020	De Oliveira A	2	Models for sim	http://link.	64	7	1063-1084	Brazil	South America	Brazil	Plant productivity	Meteorological, F	Accumulated Deg
43	2020	De Oliveira A	5	Machine learn	http://link.	64	4	671-688	Brazil	South America	Brazil	Plant productivity	Meteorological, F	Machine Learning
44	2017	Da Silva, Rica	6	Spatio-tempo	http://link.	61	5	785-795	Brazil	South America	Brazil, Australia	Insect	Meteorological, C	CLIMEX modeling
45	2015	Khalid, Bush	2	Environmenta	http://link.	59	11	1721-1746	Pakistan	Asia	Pakistan	Insect	Meteorological, H	Hotspot Analysis,
46	2002	Gagnon, Alex	3	The El Niño So	http://link.	46	2	81-89	Colombia,Ecuado	South America	Canada	Human Health, Cli	Meteorological a	Correlation and la
47	2021	Zakari, Friday	2	Comparison o	https://lin	65	7	1053-1067	Nigeria	Africa	Nigeria	Animal	Meteorological a	GraphPad Prism 6
48	2018	Zakari, Friday	5	Daily rhythms	http://link.	62	12	2231-2243	Nigeria	Africa	Nigeria	Animal	Meteorological a	Descriptive Statis
49	2015	Van Tilburg,	10	Semen variab	http://link.	59	5	561-573	Brazil	South America	Brazil	Animal	Meteorological a	STRAP and UNIV
50	2015	Rutty, Michel	2	Bioclimatic co	http://link.	59	1	37-45	Barbados, Saint L	South/North Ar	Canada	Thermal comfort	Tourism & Leisure	Descriptive Statis
51	2008	Prospero, Jo	6	Relationship b	http://link.	52	8	823-832	Barbados	North America	Barbados, USA	Human Health, Ae	Meteorological, H	Descriptive statist
52	2005	Gyan, K.; He	8	African dust c	http://link.	49	6	371-376	Trinidad	North America	Trinidad	Human Health, Ae	Meteorological, H	Poisson regressio
53	2023	Tao, Zexing	1	Predicting the	https://lin	67	1	107-119	Kazakhstan, Uzbe	Asia	China	Phenology	Modelling	Compared the spa
54	2009	Ardalan, Ali;	8	Evaluation of	http://link.	53	3	247-254	Iran	Asia	Iran	Climate (Flooding)	Qualitative (ques	Document Review
55	2023	Mallik, Piyash	2	Sub-regional	https://lin	67	10	1591-1605	India	Asia	India	Plant Productivity	Meteorological, S	Climate Model-Ba
56	2022	Kabano, Pet	3	Spatiotempor	https://lin	66	2	385-396	Uganda (Kampala	Africa	United Kingdom (Kab	Thermal comfort &	Meteorological (n	Regression analy
57	2012	Gomes, Char	2	Lightning safe	http://link.	56	6	1011-1023	Sri Lanka, India, S	Asia and Africa	Malaysia	Animal	Animal data (Obs	Case study analys
58	2009	Njuguna, Joh	3	Malaria morbi	http://link.	53	3	299-304	Kenya	Africa	Kenya (Njuguna, Muit	Human Health, Cli	Human health da	Trend analysis, Bi
59	2022	Nili, Sairan; A	6	The effect of	https://lin	66	8	1613-1626	Iran	Asia (Middle Ea	Iran (Nili, Asadgol, Dal	Human health, Cli	Meteorological d	Artificial Neural Ne
60	2022	Fitchett, Jenn	2	Exploring pub	https://lin	66	2	301-311	South Africa	Africa	South Africa (Fitchett	Human Health, Cli	Survey/question	Descriptive statist
61	2005	Prasad, V. Kr	3	Climatic contr	http://link.	50	1	6–16	India	Asia	India (Prasad, Badarir	Phenology, Climat	Meteorological d	NDVI time-series
62	2024	Aryal, Sugam	5	Drought, temp	https://lin	68	6	1093-1108	Nepal	Asia	Germany (Aryal, Grieß	Plant productivity	Plant data (stable	Correlation analys
63	2015	Zhu, Likai; Sc	3	Comparison o	http://link.	59	10	1373-1384	Zambia, Angola, N	Africa (souther	United States of Amer	Plant productivity	Satellite remote s	Time-series NDVI
64	2023	Mushimbei, M	2	Adapting to a	https://lin	67	2	253-263	Zambia	Africa	Zambia (Mushimbei),	Climate, Plant pro	Survey and qualit	Thematic and cor
65	2023	Ferreira, Nicc	9	Drought patte	https://lin	67	1	133-148	South Africa	Africa	Germany (Ferreira, Rö	Climate, Plant pro	Meteorological d	Drought indices (F
66	2020	Duarte, Yury	2	NASA/POWE	http://link.	64	3	319-329	Brazil	South America	Brazil (Duarte, Sentel	Climate, Plant pro	Meteorological d	Statistical compa
67	2020	Liang, Boyi;	6	Examining lan	https://lin	64	11	1911-1922	Brazil, Paraguay,	South America	People's Republic of	Phenology, Plant	Meteorological d	Time series analy
68	2014	Massa, Carol	3	On how Cattle	http://link.	58	9	1879-1891	Morocco, Wester	South America,	Argentina (Massa, Do	Bird, Climate	Bird distribution a	Principal compon
69	2021	Craparo, A. C	5	Warm nights d	https://lin	65	2	181-192	Tanzania	Africa	Vietnam (Craparo), Sc	Phenology, Plant	High-resolution n	Generalized addit
70	2024	Breetzke, Gre	2	The impact of	https://lin	69	10	2425-2440	South Africa	Africa	South Africa (Breetzk	Thermal comfort &	Meteorological d	Descriptive statist
71	2020	Tran, Dang N	9	Spatial patter	http://link.	64	5	863-872	Vietnam	Asia	Vietnam (Tran, Nguye	Thermal comfort &	Meteorological d	Vulnerability asse
72	2020	Pumijumnon	6	Effects of the	https://lin	64	9	1481-1495	Thailand	Asia	Thailand (Pumijumnor	Climate, Phenolog	Tree ring width in	REDFIT spectral a
73	2016	Phung, Dung	7	The spatial dis	http://link.	60	6	857-865	Vietnam	Asia	Australia (Phung, Rut	Human health, Cli	Meteorological d	Analytic Hierarchy
74	2015	Phung, Dung	9	Association b	http://link.	59	9	1321-1331	Vietnam	Asia	Australia (Phung, Hua	Human health, Cli	Meteorological d	Spline function, d
75	2024	Oliveira, Luca	5	Spatiotempor	https://lin	68	3	479-494	Brazil	South America	Brazil (Oliveira, Junior	Thermal comfort &	Meteorological d	Nonparametric Ma
76	2020	Nguyen, Huo	5	Temporal rela	http://link.	64	3	389-396	Vietnam	Asia	Australia (Chu, Ruthe	Human health, Cli	Meteorological d	Generalized linea
77	2016	Nguyen, Jen	2	Daily indoor-t	http://link.	60	2	221-229	Brazil, Vietnam	South America	USA (Nguyen, Docke	Climate, Urban &b	Meteorological d	Pearson correlati
78	2022	Mawa, Zanna	6	Life history tra	https://lin	66	5	927-943	Bangladesh	Asia	Bangladesh (Mawa, H	Fish, Climate	Meteorological d	FiSAT, Past 4.03,
79	2023	Liu, Lilingjun;	2	Analysis of he	https://lin	67	12	2055-2068	Myanmar, Laos, V	Asia	Singapore (Liu, Qin)	Climate, Human he	Meteorological d	Universal thermal
80	2019	Hurtado-Día	9	Short-term ef	http://link.	63	12	1641-1650	Mexico	South America	Mexico (Hurtado-Día	Thermal comfort &	Meteorological d	Poisson regressio
81	2018	Hinnah, Fern	4	Weather-bas	http://link.	62	10	1847-1860	Brazil	South America	Brazil (Hinnah, Sentel	Plant productivity	Meteorological d	Progress curves,
82	2015	Meza Torres,	4	Distribution of	http://link.	59	2	137-150	Argentina, Bolivia	South America	Argentina (Meza Torre	Plant productivity	Meteorological re	Hybrid Single Part
83	2022	Montes, Carl	3	Variable clima	https://lin	66	11	2237-2249	Afghanistan, Ban	Asia	Mexico (Montes), Ban	Plant productivity	Meteorological d	Generic fungal inf
84	2020	Gebregeorgi	8	Historical drou	http://link.	64	5	739-753	Ethiopia	Africa	Poland (Gebregeorgis	Climate, Phenolog	Meteorological d	Cross-dating of tr
85	2019	Diniz, Fernan	3	Valuation of th	http://link.	63	11	1507-1516	Brazil	South America	Brazil (Diniz, Iwabe, Pi	Thermal comfort &	Meteorological d	Human Thermal D
86	2024	Alencar, Jan	3	Ecological nic	https://lin	68	5	891-898	Brazil (Sao Paulo)	South America	All from Brazil	Insect/Ecology	Occurrence reco	Ecological niche
87	2018	Fitchett, Jenn	2	An analysis of	http://link.	62	12	2161-2172	South Africa	Africa	Both South African	Tourism	Climatic mentions	Manual content a
88	2018	Trifković, Julij	8	Influence of d	http://link.	62	6	1097-1108	Serbia	Central Europe	Bosnia and Herzegov	Animal, climate	Air temperature a	Mixed between-w
89	2023	Savić, Stevar	10	Hospital admi	https://lin	67	4	695-704	Novi Sad, Serbia	Central Europe	All Serbia	Human health and	Temperature Dat	Pearson correlati
90	2021	Mimić, Gorda	4	The influence	https://lin	65	6	917-928	Vojvodine, Serbia	Central Europe	All Serbia	Plant productivity	Bi-hourly airborn	Atmospheric disp
91	2023	Milošević, Dra	6	Analysis of lor	https://lin	67	6	1105-1123	Serbia	Central Europe	Serbia and Croatia	Thermal comfort	Meteorological d	RayMan Pro mode
92	2022	Milošević, Dra	8	Analysis of air	https://lin	66	2	371-384	Serbia	Central Europe	Serbia and Germany	Urban climate	Long-term air ten	Statistical analysi
93	2023	Malinović-Mil	4	Evaluation of	https://lin	67	5	807-819	Serbia	Central Europe	Serbia and Russia	Themral comfort a	Meteorological d	Calculation of the
94	2022	Janjić, Filip; B	9	Seasonal diffe	https://lin	66	4	691-698	Serbia	Central Europe	Serbia	Animal	Clinical data, hen	Kruskal-Wallis tes
95	2023	Arsenović, Da	6	The effects of	https://lin	67	10	1581-1589	Serbia	Central Europe	Serbia	Climate change ar	Daily average ten	Generalized addit
96	2009	Šikoparija, B.	7	The Pannonia	http://link.	53	3	263-272	Serbia and Maced	Central Europe	Serbia, United Kingdo	Plant productivity	Ambrosia pollen	Spearman's rank
97	2011	Ruml, Mirjana	4	Predicting ap	http://link.	55	5	723-732	Belgrade, Serbia	Central Europe	Serbia	Phenology	Phenological dat	Statistical analysi
98	2010	Ruml, Mirjana	3	Evaluation of	http://link.	54	4	411-422	Belgrade, Serbia	Central Europe	Serbia	Phenology	Phenological dat	Statistical method
99	2020	Nassiri-Maha	2	Using the Aqu	https://lin	64	12	2105-2117	Iran	Asia	Iran	Agrometeorology	Soil physicochen	AquaCrop model
100	2016	Basarin, Bilja	3	Quantificatior	http://link.	60	1	139-150	Serbia	Central Europe	Serbia and Gemrany	Human health and	Daily meteorologi	Physiologically Eq

101	2023	Nikolaou, Ge	1	Estimating cu	https://link	67	11	1745-1756	Cyprus	Middle East	Cyprus and Greece	Plant productivity	Climatic data (sol	NOVA, regression
102	2024	Nikolaou, Ge	1	Calibration an	https://link	68	1	1-15	Cyprus	Middle East	Greece, Cyrpus and It	Climate and Agricu	Climatic data (sol	Calibration and va
103	2022	Pantavou, Ka	1	Machine learn	https://link	66	10	1973-1984	Cyprus	Middle East	Cyprus and Greece	Thermal sensation	Field survey data	Machine learning
104	2009	Sabziparvar,	2	An improved e	https://link	53	3	239-245	Iran	Asia	Iran	Climate - radiation	Daily clear-sky br	Linear regression
105	2021	Chaturvedi, S	1	Understandin	https://link	65	7	1161-1175	India	Asia	India	Human health and	Meteorological da	Dynamical malaria
106	2024	Hambira, Wa	6	Perspectives	https://link	69	10	NPY	Botswana	Africa	Botswana, South Afric	Tourism	Meteorological D	Descriptive statist
107	2024	Roffe, S., Ajik	3	High aerospo	https://link	69	10	NPY	South Africa	Africa	South Africa, Nigeria,	Aerobiology	Meteorological da	Composite analys
108	2017	Burkart, Katri	1	What drives co	https://link	61	6	1073-1080	Bangladesh, India	Asia	USA	Thermal stress and	Meteorological D	Poisson regressio
109	2016	Diokhane, Ar	5	Linkages betw	https://link	60	4	557-575	Senegal	Africa	Senegal and USA	Human health	Meteorological da	Comparison of ob
110	2022	Parkes, B	1	Heat stress in	https://link	66	8	1531-1545	African countries (	Africa	UK, Switzerland and U	Heat stress and cli	Climate model da	High temporal res
111	2017	Gommes, Re	1	CropWatch ag	https://link	61	2	199-215	Botswana, sub-Sa	Global	China	Agroclimatology	Satellite-based d	Correlation analys
112	2014	Almaguer, Mi	4	Temporal dyn	https://link	58	7	1459-1470	Cuba	South America	Cuba and Spain	Aerobiology	Aerobiological da	Scheffé test for di
113	2022	Callejas, Ivan	1	Microclimate a	https://link	66	9	1877-1890	Brazil	South America	Brazil	Microclimate and T	Field monitoring (	Descriptive statist
114	2024	da Conceiçã	3	Calibration, te	https://link	68	9	1703-1716	Londrina, Paraná,	South America	Brazil	Agriculture and Cli	Experimental field	Calibration and te
115	2019	Silva, Igor Ro	4	Excess of chil	https://link	63	11	1517-1524	Brazil	South America	Brazil (all)	Human Health	Met station data,	Heat Index, HTCI,
116	2023	da Silva Sant	8	Climate suitab	https://link	67	7	1185-1197	Mexico, Puerto Ri	Carribean Island	Brazil (all)	Insect	Gridded historica	CLIMEX 4.0.2, Ec
117	2016	Algeciras, Jo	5	Human therma	https://link	60	8	1151-1164	Cuba	Carribean Island	Cuba/Spain, Spain, C	Human Health	Met station data	effective tempera
118	2020	Roncancio, D	3	Classification	https://link	64	10	1795-1806	Colombia	South America	Brazil, Cuba, Brazil	Human Health	Met station data	C++ program, freq
119	2002	Rusticucci, M	3	Association b	https://link	46	1	42-51	Argentina	South America	Argentina	Human Health	Emergency room	IRE, TRF (biomet i
120	2020	Zinat, Most. F	4	Appraising dro	https://link	64	10	1687-1697	Bangladesh	Asia	Bangladesh (all)	Phenology	Met station data,	Standardized Pre
121	2014	Yoshifuji, Nat	7	Inter-annual v	http://link.	58	9	2025-2029	Thailand	Asia	Japan, Japan, Japan,	Phenology	Sap Flow, Soil Mo	Time Series Analy
122	2013	Yahia, Mooha	2	Evaluating the	http://link.	57	4	615-630	Syria	Middle East	Sweden	Human Health	Met station data,	PET, OUT_SET, E
123	2014	Tawatsupa, B	4	The associatio	http://link.	58	2	203-215	Thailand	Asia	Thailand/Australia, Au	Human Health	Population, Morta	Statistical Analysi
124	2015	Tanaka, Nob	9	Throughfall ur	http://link.	59	9	1145-1156	Thailand	Asia	Japan, USA/Japan, Ja	Phenology	Met station data,	Leaf Area Index,
125	2011	Segnalini, Ma	6	Dynamics of th	http://link.	55	2	253-263	Mediteranean Bas	Europe	Italy	Animal	Met Station Data	THI index, maps
126	2001	Schreiber, K.	1	An investigatio	http://link.	45	2	81-89	Puerto Rico	Carribean Island	USA	Insect, Human Hea	CDC Health Data	Thorthwaite-Math
127	2023	Ponsen, Sirip	3	The influence	https://link	67	4	705-716	Thailand	Asia	Thailand	Insect	Soil and animal m	Staitistical Analys
128	2014	Ohta, Shunji;	2	Effect of irriga	http://link.	58	3	349-359	India	Asia	Japan	Insect	1 year of mosquito	ECD-mg model
129	2013	Khongdee, T	3	The effects of	http://link.	57	3	349-354	Thailand	Asia	Thailand	Animal	Met station data,	Statistical Analysi
130	2006	Khongdee, S	5	Effects of eva	http://link.	50	5	253-257	Thailand	Asia	Thailand, Thailand, Au	Animal	Animal observatio	t-test using PROC
131	2014	Jian, Wang; D	4	Differences of	http://link.	58	6	1087-1094	Thailand	Asia	Thailand	Animal	Animal skin samp	Analysis of skin sa
132	2023	Jayaramu, Ve	10	Leptospirosis	https://link	63	3	423-437	Malaysia	Asia	Malaysia, Malaysia, UK	Animal and Human	Leptospirosis cas	hydrometeorologi
133	2017	Gilioli, Gianni	11	A temperature	http://link.	61	11	1899-1911	Southeast Asia, S	Asia	Italy, Italy, Argentina, S	Animal	Met station data,	Population dynam
134	2001	De Dear, R.; S	2	The adaptive	http://link.	45	2	100-108	Pakistan, Indones	Asia	Australia, USA	Urban and Building	In situ meteorlogic	ASHRAE
135	2024	Conde-Gutié	4	Parallel predic	https://link	68	6	1043-1060	Mexico	North America	Mexico	Human Health	Met station data,	artificial neural ne
136	2013	Aengwanich,	2	Effect of polyp	http://link.	57	1	137-143	Thailand	Asia	Thailand	Animal	Animal Data	Data with treatmen
137	2019	Aboubakri, O	4	The impact of	http://link.	63	9	1139-1149	Iran	Middle East	Iran (all)	Human Health	Burden of Diseas	Poisson and linea
138	2018	Magalhães, S	4	Determining th	http://link.	62	7	1187-1197	Brazil	South America	Brazil, Canada, Brazil	Phenology	Optical phenolog	leaf area index (LA
139	2018	Macías-Cruz	6	Thermoregula	http://link.	62	12	2151-2160	Mexico	North America	Mexico (all)	Animal	Animal observatio	Statistical analysi
140	2018	López, E.; Me	7	Stress-related	http://link.	62	4	493-500	Mexico	North America	Mexico (all)	Animal	Animal observatio	Models and linear
141	2019	Locosselli, G	5	Impact of tem	http://link.	63	12	1683-1692	Brazil	South America	Brazil, Germany, Ger	Phenology	Plant samples, tre	Statistical analysis
142	2018	Kjellstrom, To	5	Estimating po	http://link.	62	3	291-300	Whole globe	Whole World	Cypress/Australia/New	Human Health	Gridded climate d	Wet Bulb Globe Te
143	2024	González-Mo	6	Seasonal and	https://link	68	7	1421-1435	Mexico	North America	Mexico, Mexico, Spain	Animal	Lizard observatio	Ordinary Least Sq
144	2017	Chmura, Paw	6	Physical activ	http://link.	61	4	677-684	Brazil	South America	Poland	Human Bioemeter	Human Health, M	Grouping of envir
145	2018	Calderón-Ez	6	Behaviour of C	http://link.	62	9	1721-1732	Mexico	North America	Mexico and Spain	Phenology	Pollen Sampling,	Statistical Analysi
146	2017	Azcárate, T.;	2	Influence of g	http://link.	61	9	1585-1592	Mexico	North America	Mexico	Human Bioemeter	Arterial pressure	Bivariate, Superp
147	2024	Avendaño-R	10	Effects of pari	https://link	68	3	427-434	Mexico	North America	Mexico	Animal	Animal data (colo	General Linear Mo
148	2022	Pompa-Garc	4	Climate sensit	https://link	66	8	1711-1723	Mexico	North America	Mexico and Spain	Phenology	Plant sampling, g	Tree ring analysis
149	2023	Ortega-Rosa	6	Five-year airb	https://link	67	11	1853-1868	Mexico	North America	Mexico	Phenology, Huma	Sampling airborn	Pollen calendar c
150	2024	Craig, Christo	2	Forecasting th	https://link	68	12	2731-2742	Mexico, US, Cana	North America	USA	Human, Tourism	UTCI, historical cl	Forecasting mode
151	2019	Zuhra, Syeda	3	Appraisal of th	http://link.	63	12	1669-1682	Pakistan	Asia	Pakistan	Human, Health	Meteorological da	Principal Compon
152	2020	Zhang, Ruibo	10	Climatic chan	http://link.	64	5	841-851	Kazakhstan	Asia	China	Phenology	Samples of pre-tr	Statistical Analysi
153	2016	Yadav, Vijay	9	Expression an	http://link.	60	3	381-389	India	Asia	India	Animal	Animal data (Biol	Statistical compar
154	2022	Yaghoubi, Fa	3	Changes in sp	https://link	66	3	431-446	Iran	Asia	Iran	Agricultural	Gridded AgMERR	Trend analysis, de
155	2020	Yaghoubi, Fa	3	Performance	https://link	64	9	1519-1537	Iran	Asia	Iran	Agricultural	Gridded AgMERR	Weather data eva
156	2018	Yahia, Mooha	5	Effect of urba	http://link.	62	3	373-385	Tanzania	Africa	Sweden	Thermal comfort a	Simulation proce	Numerical microcl
157	2018	Yakubu, A.; O	3	Predicting hea	http://link.	62	7	1181-1186	Nigeria	Africa	Nigeria	Animal	Heat tolerance tra	Automatic linear r
158	2022	Yasuoka, Jun	7	Microclimate c	https://link	66	1	45-54	Brazil	South America	Brazil	Agricultural, Micro	Experimental	Treatment of cano
159	2023	Rauf, Zahid;	11	The Western H	https://link	67	11	1477-1492	Pakistan	Asia	Pakistan and People's	Phenology	Gridded climate d	Tree ring width ch
160	2019	Zarei, Abdol R	2	Modified versi	http://link.	63	7	911-925	Iran	Asia	Iran	Agricultural	Meteorological da	Effective precipita
161	2021	Zein, Sima; A	11	Influence of g	https://link	65	12	2053-2057	Kyrgyzstan	Asia	Jordan, Kyrgyzstan an	Human	Human data	Statistical analysis
162	2016	El Zerey-Bela	2	Morphologica	http://link.	60	4	577-589	Algeria	Africa	Algeria	Phenology	Meteorological da	Statistical analysis
163	2004	Barradas, V	3	Stomatal cond	http://link.	48	3	119-127	Mexico	North America	Mexico	Phenology	Plant data	Measurement of s
164	2005	Borchert, Rol	4	Phenology of	http://link.	50	1	57-65	Mexico and Centra	North America	USA, Mexico	Phenology	Phenological obs	Modeling bud brea
165	2005	O'Neill, Marie	5	Impact of con	http://link.	50	2	121-129	Mexico	North America	USA, London, and Me	Human	Air and weather d	Robust Poisson re
166	2008	Gómez-Mend	6	Assessing ons	http://link.	52	6	511-520	Mexico	North America	Mexico	Phenology	NDVI data, Meteo	Temporal and spa
167	2010	Bendix, Jörg;	7	Model parame	http://link.	54	3	283-295	Ecuador	South America	Germany	Phenology	Meteorological da	Decomposition of
168	2010	Gonçalves, F	7	Indoor and ou	http://link.	54	4	347-355	Brazil	South America	Brazil and Austria	Human and Pheno	Indoor and outdo	Total spore count
169	2013	Do Prado Pai	11	Thermograph	http://link.	57	1	59-66	Brazil	South America	Brazil and Austria	Animal	Physiological & In	Descriptive Statist
170	2014	Shrestha, A.	8	Larger antelop	http://link.	58	1	41-49	South Africa	Africa	The Netherlands and	Animal	Implantation of a	Daily and seasona
171	2015	Katiyatiya, C.	3	Seasonal vari	http://link.	59	6	733-743	South Africa	Africa	South Africa	Animal Biometeoro	Animal Data, Met	Generalised Linea
172	2015	Bosco, Leosa	6	Apple produc	http://link.	59	7	773-782	Brazil	South America	Brazil	Plant Biometeorolo	Plant Data	Analysis of varian
173	2015	Idder-Ighili, H	4	Modeling the	http://link.	59	10	1425-1436	Algeria	Africa	Algeria	Plant Biometeorolo	Meteorological D	analysis of varian
174	2016	Savage, M. J	1	Nowcasting d	http://link.	60	2	183-194	South Africa, USA	Africa and North	South Africa	Climate	Agrometeorologic	Nowcast Modellin
175	2017	Fourment, Me	6	Tannat grape	http://link.	61	9	1617-1628	Uruguay	South America	Uruguay, France	Plant Biometeorolo	Meteorological D	ANOVA, principal
176	2017	Antivilo, Fran	6	Macro- and m	http://link.	61	12	2033-2045	Argentina, USA	South America,	Argentina, USA	Plant Biometeorolo	Meteorological D	Generalized linea
177	2017	Katiyatiya, C.	2	Hair coat cha	http://link.	61	12	2183-2194	South Africa	Africa	South Africa	Animal Biometeoro	Animal Data, Met	PROC GLM of SA
178	2018	Benmoussa,	4	Climate chang	http://link.	62	12	2245-2255	Tunisia	Africa	Tunisia, Germany	Plant Biometeorolo	Meteorological D	R package chillR,
179	2019	Müller, Andre	6	Neotropical fe	http://link.	63	10	1393-1404	Brazil	South America	Brazil	Phenology	Plant Data, mete	Generalized Additi
180	2021	Rezaei, Ehsa	6	The use of rer	http://link.	65	4	565-576	South Africa	Africa	Germany	Plant Biometeorolo	Remotely sensed	MODIS MCD12Q2
181	2022	Snyman, Pas	2	Phenological	https://link	66	4	699-717	South Africa	Africa	South Africa	Phenology	meteorological da	Linear regression
182	2022	Potgieter, An	4	The associatio	https://link	66	5	865-874	South Africa	Africa	South Africa	Human Biometeorol	meteorological da	distributed lag nor
183	2022	Deihimfard, R	4	Increased hea	https://link	66	7	1365-1378	Iran	Asia	Iran	Plant Biometeorolo	Climate data, soil	SPAW (soil–plant-
184	2024	Silveira, Robs	5	Are there diffe	https://link	68	2	229-236	Brazil	South America	Brazil	Animal Biometeoro	Animal data, Envi	Levene test, ANO
185	2024	Kapwata, Tha	6	Identifying hea	https://link	68	2	381-392	South Africa	Africa	South Africa, USA, Ita	Human Biometoro	Mortality data, po	Distributed lag nor
186	2024	Strydom, Lar	7	Mapping tree	https://link	69	10	2461-2474	South Africa	Africa	South Africa, USA	Thermal Comfort	LiDAR data, weat	NicheMapR biophy
187	2000	Pochron, Sha	1	Sun avoidanc	http://link.	44	3	141-147	Tanzania	Africa	USA	Animal Biometeoro	Meteorological da	linear statistical m
188	2020	Talhi, Assia;	4	Towards a pre	http://link.	64	4	651-662	Algeria	Africa	Algeria, France	Thermal Comfort	Objectve and Su	Correlation analys
189	2017	Tamerius, Ja	7	Influenza tran	http://link.	61	4	613-622	Nicaragua	North America	Nicaragua, USA	Human Biometeor	Human laborator	Chow Forecast Te
190	2018	Tapak, Leili;	5	Investigating t	http://link.	62	12	2109-2118	Iran	Asia	Iran	Human Biometeor	Meteorological D	Random forest reg
191	2017	El-Tarabany,	3	Physiological	http://link.	61	1	61-68	Egypt	Africa	Egypt	Animal Biometeoro	Animal Data, Met	statistical system
192	2024	Teixeira, Anto	6	Monitoring an	https://link	68	12	2597-2612	Brazil	South America	Brazil	Climate	Gridded weather	geographic inform
193	2023	Ingty, Tenzin	5	Climate chang	https://link	67	5	913-925	India	Asia	USA, India	Phenology	remotely sensed	Remote sensing t
194	2018	Texier, N.; De	13	Spatio-tempo	http://link.	62	11	1931-1944	Cameroon	Africa	Belgium, Cameroon, U	Phenology	Plant data, Gridd	principal compone
195	2020	Thapliyal, Ga	7	First record of	https://link	64	9	1629-1634	India	Asia	India	Phenology	Plant Data, Mete	Not stated in this s
196	2020	Tharranum, A	3	Evaluation of t	http://link.	64	3	445-460	India	Asia	India	Plant productivity	apterous mustard	Modelling
197	2013	Titto, Cristian	6	Effects of an e	http://link.	57	2	299-306	Brazil	South America	Brazil, Portugal	Animal Biometeoro	Weather data, an	ANOVA, Student'
198	2024	Tiwari, Manis	5	Hypoxia relate	https://link	68	8	1675-1687	India	Asia	India	Animal Biometeoro	Animal data	ANOVA, Tukey's
199	2012	Tochihara, Y	6	The use of lan	http://link.	56	6	1055-1064	Indonesia, Japan,	Asia, Europe	Japan, UK, Indonesia	Thermal Comfort	Survey data (que	Descriptive analys
200	2015	Torki, Mehrar	3	Single and co	http://link.	59	9	1169-1177	Iran	Asia (Middle Eas	Iran	Animal Biometeoro	Animal productiv	Statistical Analysi

201	2015	Torki, Mehrar	4	Response of I	http://link.	59	5	575-584	Iran	Asia (Middle Ea:	Iran	Animal Biometeor	Animal data (feec	Statistical analysi:
202	2024	Torsoni, Guill	6	Climatic zonin	https://linl	68	5	979-990	Brazil, Argentina, I	South America	Brazil	Plant, Climate Bior	Meteorological da	WorldClim 2.1 platt
203	2024	Da Costa Tra	10	Analysis of the	https://linl	68	3	463-477	Brazil	South America	Brazil	Climate Biometeor	Meteorological D	Bimeteorological i
204	2023	Tripathi, Utka	9	Seasonal and	https://linl	67	2	311-320	India	South Asia	India	Animal , Climate Bi	Meteorological da	one-way ANOVA
205	2024	Upadhyay, V	4	Deciphering tl	https://linl	68	9	1885-1895	India	South Asia	India	Animal, Climate Bi	Microclimate data	Statistical Analysi
206	2021	Valeriano, Ta	6	A process-ba	https://linl	65	12	2037-2051	Brazil, India, Austr	South America,	Italy, Brazil, Luxembou	Plant Biometeorol	Plant data, Metec	ARISE Model
207	2020	Valizadeh, M	2	An evaluation	http://link.	64	1	29-37	Iran	Asia	Iran	Climate Biometeor	Meteorological da	Outdoor Tourism I
208	2004	Valtorta, Silvi	2	Evaporative c	http://link.	48	4	213-217	Argentina	South America	Argentina	Animal Biometerol	Animal data, Met	Experimental mod
209	2006	Valtorta, S. E	6	Daily rhythms	http://link.	50	3	133-138	Argentina	South America	Argentina	Animal Biometerol	Animal data, Met	Statustical analys
210	2008	Valtorta, Silvi	8	Water salinity	http://link.	52	3	239-247	Argentina	South America	Argentina	Animal Biometerol	Animal data, Met	Statistical Analysi
211	2010	Vani, R.; Shiv	3	Oxidative stre	http://link.	54	5	553-562	India	South Asia	India	Animal Biometerol	Animal data	Statistical analysi:
212	2016	Hosseini-Vas	3	Growth, immu	http://link.	60	8	1183-1192	Iran	Asia	Iran	Animal Biometerol	Animal data	Statistical analysi:
213	2023	Vashisth, Ana	2	Prediction of r	https://linl	67	3	539-551	India	South Asia	India	Plant Biometeorol	Meteorological da	Models (Machine
214	2024	Vasisth, Rasl	10	Selection of re	https://linl	68	7	1397-1409	India	Asia	India	Animal Biometeor	Meteorological da	Statistical analysi:
215	2021	Verma, Nitika	12	Comparative (	https://linl	65	7	1025-1032	India	Asia	India	Animal Biometeor	Animal Data	Statistical Analysi
216	2023	Verma, K. K.;	5	Heat ameliora	https://linl	67	6	1063-1076	India	Asia	India	Animal, Climate Bi	Animal Data (Phy	Statistical analysi:
217	2021	Verón, Gusta	8	Association b	https://linl	65	8	1399-1414	Argentina	South America	Argentina	Human, Climate Bi	Human Health, M	Statistical Analysi
218	2019	Vieira, Frede	8	Impact of exp	http://link.	63	6	777-785	Brazil	South America	Brazil	Animal Biometeor	Animal Data	Statistical Analysi
219	2015	Vourlitis, Gec	4	Variations in e	http://link.	59	2	217-230	Brazil	South America	Brazil, USA	Climate Biometeor	Meteorolical data	Statistical Analysi
220	2015	Wagner-Ridd	7	Determining tl	https://linl	59	10	1499-1509	Brazil	South America	Brazil, Canada	Climate biometeor	Meteorological da	Statistical Analysi
221	2016	Waiz, Syma A	7	Heat stress ar	http://link.	60	9	1357-1366	India	Asia	India	Animal Biometeor	Animal data	Statistical analysi:
222	2011	Wakabayash	6	Comparison o	http://link.	55	4	509-517	Malaysia	Asia	Japan, Malaysia	human, climate bi	Human data	statistical analysis
223	2014	Wanner, Sam	5	Physical exer	http://link.	58	6	1077-1085	Brazil	South America	Brazil	Animal, Climate Bi	Animal Data	Statistical Analysi
224	2022	Weitz, Charle	3	Individually ex	https://linl	66	6	1145-1162	India	Asia	USA, India	Human, Climate Bi	Meteorlogical dat	Statistical analysi:
225	2011	Wijayanto, Ti	6	Comparison o	http://link.	55	4	491-500	Malaysia	South East Asia	Japan, Malaysia	Thermal comfort, I	Human health da	SPSS statistical a
226	2023	Wijerathna, T	2	Time series ar	https://linl	67	2	275-284	Sri Lanka	South Asia	Sri Lanka	Human health	Human health an	Univariate ARIMA
227	2024	Gayathri, S. L	3	Advancing ma	https://linl	68	11	2253-2265	India	South Asia	India	Animal	Meteorological da	California mastitis
228	2018	Sabzevari, A:	5	Evaluating the	http://link.	62	7	1135-1145	Iran	Middle East	Iran	Tourism	Meteorological da	TCI, PMV, and CIT
229	2022	Safari, Zahra	3	Health impact	https://linl	66	9	1891-1902	Iran	Middle East	Iran and Italy	Human health	Air pollution data	BenMAP-CE mod
230	2021	Sahoo, Prafu	5	Pre-to-post lc	https://linl	65	2	205-222	India	South Asia	India and Brazil	Human health	Air pollutant data	Kendall rank corre
231	2022	Sakhri, Hadjir	3	Study on corr	https://linl	66	12	2517-2528	Algeria	North Africa	Algeria and UK	Thermal comfort, u	Human behaviou	Psychological eva
232	2015	Salas-Morale	3	The relationsh	http://link.	59	12	1861-1874	Mexico	North America	Mexico	Plant productivity	Meteorological da	Statistical analyse
233	2019	Salehnia, Na	5	Climate data c	http://link.	63	7	861-872	Iran	Middle East	Iran	Plant productivity	Meteorological da	Data mining (clust
234	2023	Salvian, Maya	11	Heat stress or	https://linl	67	2	347-354	Brazil	South America	Brazil	Animal, heat stress	Animal data and	Climate indices ar
235	2019	Samad, H. A.	12	Alpha lipoic ac	http://link.	63	10	1331-1346	India	South Asia	India	Heat stress, anima	Animal data and	DNA testing and t
236	2011	Sanches, Lu	5	Performance	http://link.	55	3	303-312	Brazil	South America	Brazil	Plant productivity	Meteorological da	Paired t-test and :
237	2021	Santos, Abra	8	Wet and warm	https://linl	65	2	247-256	Brazil	South America	Brazil	Plant productivity	Insect data	Climatic indices
238	2017	Santos, Seve	8	The use of sir	http://link.	61	2	217-225	Brazil	South America	Brazil	Heat stress, anima	Animal data and	Statistical analyse
239	2022	Chandran M.	5	Evaluating the	https://linl	66	1	55-69	India	South Asia	India	Plant productivity	Plant data and m	Sequence analys
240	2019	Sarkar, Uttan	11	Climato-envir	http://link.	63	8	991-1004	India	South Asia	India and Czech Repu	Phenology, fish	Animal data and	Statistical analyse
241	2022	Sarkar, Uttan	8	Inland heat wa	https://linl	66	1	235-245	India	South Asia	India	Heat stress, hydro	Meteorological da	Inland heat wave i
242	2022	Sarkar, Uttan	9	Combining sta	https://linl	66	7	1415-1427	India	South Asia	India	Climate	Meteorological da	Vulnerability index
243	2023	Kakarla, Saty	8	Weather integ	https://linl	67	2	285-297	India	South Asia	India	Human health	Meteorological da	VAR, SVR, GBM,
244	2017	Saxena, Vijay	3	Molecular cha	http://link.	61	4	701-707	India	South Asia	India	Animal	Animal data	DNA analysis
245	2012	Scarpare, Fá	5	Growing degr	http://link.	56	5	823-830	Brazil	South America	Brazil	Heat stress, plant	Meteorological da	Statistical analysi:
246	2014	Scheibler, Ell	3	Lunar and ten	http://link.	58	8	1769-1778	Mongolia	East Asia	Germany	Animal	Meteorological da	Radio Frequency
247	2023	Schmitt Filho	6	Applied nucle	https://linl	67	7	1199-1212	Brazil	South America	Brazil, USA	Animal	Animal data and	Statistical analyse
248	2004	Scian, Beatri	1	Environmenta	http://link.	48	4	206-212	Argentina	Argentina	Argentina	Phenology	Plant data	Palmer drought se
249	2017	Sedghy, Farr	6	Impact of traff	http://link.	61	1	1-9	Iran	Asia	Iran	Phenology	Plant and weathe	Statistical analysi:
250	2010	Sejian, Veera	3	Adaptive capa	http://link.	54	6	653-661	India	India	India	Animal	Animal and clima	Correlation coeffic
251	2012	Sejian, Veera	3	Effect of walki	http://link.	56	2	243-252	India	India	India	Animal	Animal and therm	data were analyze
252	2023	Roy, Shouras	1	Spatial patter	https://linl	67	1	157-164	India	India	USA	HUman health	Rainfall data	Pearson correlati
253	2004	Sentelhas, P	3	Electronic lea	http://link.	48	4	202-205	Brazil	Brazil	Brazil and Canada	Plant	Climate data	Regression analy:
254	2014	Sepehrimane	6	Impact of 900	http://link.	58	7	1657-1663	Iran	Iran	Iran	Animal	Animal and clima	SPSS statistical p
255	2018	Shahid, Moha	7	Boron applica	http://link.	62	8	1375-1387	India	India	India	Plant	Plant and Climate	SAS statistical pa
256	2015	Shakir, Muha	2	Seasonal abu	http://link.	59	5	605-616	Pakistan	Pakistan	Pakistan	Plant	Rainfall and anim	basic parameters
257	2023	Sharafi, Milac	4	Evaluation of /	https://linl	67	4	621-632	Iran	Iran	Iran	Plant	Rainfall and plant	SVR-FFA model,
258	2023	Sharma, Suti	6	A comparative	https://linl	67	10	1619-1628	India	India	India	Animal	Animal and Envirc	Pairwise comparis
259	2023	Sharma, Sak	3	Metatranscrip	https://linl	67	11	1803-1811	India	India	India	Human Health	Plant waste	CLC Genomics wo
260	2020	Sharmin, Tan	2	Effects of mic	https://linl	64	2	187-203	Bangladesh	Bangladesh	Cambridge	Human Health	Climate date	Thermal sensatior
261	2023	Shashank, C	7	Comparative a	https://linl	67	9	1435-1450	India	India	India	Animal	Animal and Clima	general linear mo
262	2022	Simath, Shifa	2	Urban therma	https://linl	66	9	1865-1876	Sri lanka	Sri lanka	UK	Human Health	Climate data	UTCI
263	2016	Shilja, Shaji;	8	Adaptive capa	http://link.	60	9	1311-1323	India	India	India	Animal	Climate and Anim	general linear moc
264	2015	Shiukhy, Sae	3	Colored plasti	http://link.	59	8	1061-1066	Iran	Iran	Iran	Plant	Climate and plant	SNK post hoc test
265	2024	Simon, Alois;	8	The interior cli	https://linl	68	4	719-730	Argentina	Argentina	Germany	Plant	Forest and Climat	R package
266	2021	Singh, Rn; Mu	7	Interactive eff	https://linl	65	11	1939-1952	India	India	India	Plant	Plant and climate	SAS software pac
267	2023	Singh, Kanika	6	Association o	https://linl	67	12	1975-1989	India	India	India	Human Health	Climate data and	GraphPad Prism :
268	2016	Soares-Colle	3	An agro-clima	http://link.	60	6	891-905	Brazil	Brazil	Brazil and USA	Plant	Plant data	multivariate linear
269	2016	Son, Ji-Youn	5	The impact of	http://link.	60	1	113-121	Brazil	Brazil	USA and Brazil	Human Health	Climate and Hum	Bayesian hierarch
270	2023	Sridhara, Sha	7	Evaluation of	https://linl	67	1	165-180	India	India	India and Germany	Animal	Animal and weath	SPSS software
271	2011	Staggemeier	2	Reproductive	http://link.	55	6	843-854	Brazil	Brazil	Brazil	Plant	Plant and climate	Rayleigh test
272	2009	Stoupel, Elya	6	Traffic accide	http://link.	53	6	523-534	Isreal	isreal	Isreal	Human	Traffic and climat	Parametric statisti
273	2018	Stürmer, Mor	6	Relationship t	http://link.	62	9	1663-1674	Brazil	South America	Brazil	Animal biometeorc	Monthly milk com	Canonical correla
274	2015	Subash, N.; (	4	Identification	http://link.	59	1	65-78	India	South Asia, Asi	India	Agricultural	Meteorological da	Mann-Kendall no
275	2013	Sudharsan, I	10	Evaluation of	http://link.	57	1	107-123	India	South Asia, Asi	India, Japan	Agricultural	Historical climate	Weather-based y
276	2020	Sulzbach, Jé	5	Evaluation of	http://link.	64	9	1473-1479	Brazil	South America	Brazil	Animal biometeorc	On site experimei	Comparative stud
277	2012	Ochiai, Ange	8	Atmospheric c	http://link.	56	4	661-667	Brazil	South America	Brazil	Health biometeorc	Meteorological da	Descriptive statist
278	2018	Oguntunde, I	3	Relationship t	http://link.	62	3	459-469	Nigeria	West Africa, Afr	Nigeria, Germany	Agricultural	Meteorological da	Descriptive statist
279	2024	Ojha, Saurab	2	Investigating t	https://linl	68	7	1451-1464	India	South Asia, Asi	India	Urban and environ	Meteorological da	Field surveys and
280	2007	Sentelhas, P	3	Leaf wetness	http://link.	51	4	265-273	Brazil (GS) and Ca	South America	Brazil and Canada	Agricultural	Field sensor data	Descriptive statist
281	2019	Oliveira, Stef	5	Effects of sha	http://link.	63	11	1465-1474	Brazil	South America	Brazil	Animal biometeorc	Meteorological c	Principal compon
282	2022	Oroud, Ibrahi	1	Integration of	https://linl	66	11	2273-2285	Jordan	Middle East	Jordan	Urban and environ	Meteorological da	GIS spatial interpc
283	2021	Othman, Nur	5	Field study of	https://linl	65	4	453-477	Malaysia	Southeast Asia	Malaysia, Japan	Human biometeor	On-site micromet	Field experiment,
284	2008	Paaijmans, K	3	A simplified m	http://link.	52	8	797-803	Kenya	East Africa, Afri	Kenya, The Netherlan	Environmental	Temperature mea	Development and
285	2008	Paaijmans, K	4	The effect of	http://link.	52	8	747-753	Kenya	East Africa, Afri	Kenya, The Netherlan	Environmental	Field experiment	Empirical compari
286	2018	Méndez-Láz	7	Climate chang	http://link.	62	5	699-707	Puerto Rico	Carribean, Nortl	Puerto Rico	Health biometeorc	Meteorological da	Poisson regressio
287	2018	Méndez-Láz	5	A heat vulnera	http://link.	62	5	709-722	Puerto Rico	Carribean, Nortl	Puerto Rico, USA	Health biometeorc	Landsat 8 TIRS re	Converting variab
288	2024	Pacheco, Dh	2	Monitoring ag	https://linl	68	10	2069-2082	Brazil	South America	Brazil	Agricultural	MODIS satellite d	Calculation of fou
289	2022	Parihar, Ashc	7	Characterizin	https://linl	66	6	1267-1281	India	South Asia, Asi	India	Agricultural	Field experimenta	Correlation analys
290	2024	Parihar, Ruch	5	Relative impo	https://linl	68	3	495-509	Tanzania	East Africa, Afri	Tanzania, Norway	Health biometeorc	Meteorological da	Sensitivity analysi
291	2024	Parihar, Ashc	26	Multi-location	https://linl	68	10	1973-1987	India	South Asia, Asi	India	Agricultural	Multi-year field tri	AMMI (Additive Ma
292	2018	Parisi, S. G.;	4	Modeling and	http://link.	62	5	883-895	Ethiopia	East Africa, Afri	Italy	Agricultural	Meteorological da	Mechanistic simul
293	2022	Paschapur, A	5	Insect pest sc	https://linl	66	7	1445-1460	India	South Asia, Asi	India	Agricultural	Field survey data	Regular monthly fi
294	2020	Passini, Robe	5	Thermal envir	https://linl	64	11	1947-1955	Brazil	South America	Brazil	Animal biometeorc	Field experiment	Analysis of varian
295	2021	Patel, N. R.; F	4	Dynamics of C	https://linl	65	7	1069-1084	India	South Asia, Asi	India	Agricultural	Eddy covariance	EC flux tower data
296	2024	Patra, Sandip	8	Influence of sl	https://linl	68	11	2241-2251	India	South Asia, Asi	India	Agricultural	Field experimenta	Generalized Linea
297	2012	Pezzopane, .	4	Agrometeorol	http://link.	56	5	843-851	Brazil	South America	Brazil	Agricultural	Field-based phe	Logistic modeling
298	2013	Pezzopane, .	5	Simple agrom	http://link.	58	7	1479-1487	Brazil	South America	Brazil	Agricultural	Field trial data ac	Univariate and mu
299	2011	Pinto-Júnior,	5	Leaf area inde	http://link.	55	2	109-118	Brazil	South America	Brazil	Forest	Field-based radia	Three methods fo
300	2018	Piovesan, Mó	5	Phenological	http://link.	62	3	413-422	Brazil	South America	Brazil, USA	Agricultural	Light trap data (1	Generalized Linea

301	2019	Piticar, Adriai	1	Changes in ag	http://link.	63	4	499-510	Chile	South America	Romania	Agricultural	Daily max/min ten	Mann–Kendall tre
302	2022	Podder, Mon	6	Physiological	https://link	66	7	1349-1364	India	Asia	India	Animal Biometeor	Field monitoring (	Statistical Analysi
303	2015	Pompa-Garc	3	Tree growth re	http://link.	59	1	89-97	Mexico	North America	Mexico	Plant Biometeorol	Sampling through	Correlation, Scatt
304	2024	Prasad, P. S.	2	Assessment c	https://link	68	9	1857-1870	India	Asia	India	Human Biometeor	Satellite Imagery,	Descriptive Statist
305	2015	Qaid, Adeb; (	2	Effect of asym	http://link.	59	6	657-677	Malaysia	Asia	Malaysia	Human Biometeor	Field monitoring t	Simulation validat
306	2024	Rafique, Rizw	5	Developing a	https://link	68	8	1587-1601	Pakistan	Asia	Pakistan, USA	Phenology	Onsite air temper	Growing Degree D
307	2023	Rafique, Rizw	6	Seasonal vari	https://link	67	5	745-759	Pakistan	Asia	Pakistan, USA	Phenology	Long term historic	Growing Degree D
308	2023	Rafique, Rizw	4	Temperature v	https://link	67	11	1881-1896	Pakistan	Asia	Pakistan	Phenology	Onsite air temper	Frequency distribu
309	2024	Rahmati, Sho	8	Risk of cardio	https://link	68	8	1689-1698	Iran	Asia	Iran, China	Human Biometeor	Satellite data, ho	Two stage regress
310	2020	Rahul, Aditya	3	Impact of gan	https://link	64	11	1933-1945	India	Asia	India	Human Biometeor	PET, UTCI, ENVI-	RMSE, R sq value
311	2005	Raj, Shammi;	4	Relationship b	http://link.	49	3	189-196	India	Asia	India	Agricultural Biome	Field observation	Frequency distribu
312	2011	Rakocevic, M	2	Time series in	http://link.	55	2	161-171	Brazil	South America	Brazil	Plant Biometeorol	Meteorological da	Generalised Poiss
313	2013	Ranjitkar, Sai	4	Flowering phe	http://link.	57	2	225-240	China and Nepal	Asia	Nepal, China, Kenya	Phenology	Meteorological da	Average peak flov
314	2020	Raoufi, Roxa	2	Simulation of t	https://link	64	10	1657-1673	Iran	Asia	Iran	Agricultural Biome	Meteorological da	Aquacrop Crop G
315	2019	Rashamol, V.	6	Effect of heat	http://link.	63	8	1005-1013	India	Asia	India	Animal Biometeor	Field observation	Decsriptive Statist
316	2017	Rathinasamy	5	Investigation c	http://link.	61	10	1709-1721	India	Asia	India, Canada	Remote Sensing	Satellite imagerie	Image analysis, sc
317	2022	Rawash, Rae	7	Expression pr	https://link	66	10	2047-2053	Egypt	Africa	Egypt	Animal Biometeor	Blood sample col	Generalised Linea
318	2024	Requia, Wee	2	Maternal expo	https://link	68	11	2297-2305	Brazil	South America	Brazil, USA	Human Biometeor	Health and morta	Two stage case c
319	2023	Rezaee, Rez	8	Summer heat	https://link	67	12	2081-2091	Iran	Asia	Iran, China	Human Biometeor	Long term historic	Distributed lag no
320	2016	Ríos, B.; Torr	6	Diurnal variati	http://link.	60	5	771-787	Mexico City	North America	Mexico	Human Biometeor	Field data from ol	Frequency distribu
321	2015	Da Silva, Jam	6	Thermal comf	http://link.	59	9	1261-1267	Brazil	South America	Brazil	Animal Biometeor	Observation and	Descriptive Statist
322	2016	Rodríguez Al	5	Human therm	http://link.	60	8	1151-1164	Cuba	North America	Cuba, Spain, German	Human Biometeor	Airport weather d	Frequency distribu
323	2006	Roig, F. A.; S	3	Stable oxyger	http://link.	51	2	97-105	Argentina	South America	Argentina, Switzerlan	Plant Biometeorol	Wood Samples	Descriptive Statist
324	2018	Romanello, N	12	Thermoregula	http://link.	62	9	1575-1586	Brazil	South America	Brazil	Animal Biometeor	Automatic weath	Generalized Linea
325	2024	Romanello, N	7	Effect of acce	https://link	68	11	2431-2446	Brazil	South America	Brazil, Italy	Animal Biometeor	Onsite weather st	Descriptive statist
326	2018	Roshan, Gho	3	Assessment c	http://link.	62	4	525-542	Iran	Asia	Iran, China, Poland	Human Biometeor	Weather station c	Hierarchical Clust
327	2016	Roshan, Gho	3	Long-term tre	http://link.	60	1	33-52	Iran	Asia	Iran, South Africa	Human Biometeor	Monthly climate d	Man Kendall Test
328	2020	Rubio, Sebas	2	Testing the Fe	https://link	64	8	1401-1408	Chile	South America	Chile	Plant Biometeorol	Automatic weath	Frequency Distrib
329	2013	Jarlan, L.; Ab	10	Linkages betw	http://link.	58	7	1489–1502	Morocco	Africa	Morocco	Agriculture Biomet	Synoptic climate	Frequency Distrib
330	2012	Laaboudi, Ab	3	Neural networ	http://link.	56	5	831-841	Algeria	Africa	Algeria	Hydrology	Meteorological S	Three layer feed f
331	2022	Lamichaney,	6	Implications o	https://link	66	12	2425-2431	India	Asia	India	Agriculture Biomet	Field monitoring a	t-test, frequency d
332	2018	Lashkari, A.;	6	Evaluation of	http://link.	62	8	1543-1556	Iran	Asia	Author one = China; A	Plant biometeorol	Gridded daily rain	Pairwise comparis
333	2013	Latha, R.; Mu	2	Response of	http://link.	57	4	645-648	India	Asia	Author one & two = In	Plant biometeorol	One observation	Time series plots o
334	2009	Lee, Jeong-I	5	Tropical Malay	http://link.	53	2	149-157	Republic of Korea	Asia	Author one = Republi	Human biometeorl	Data collected fro	Descriptive statist
335	2018	Leite, Jacina	8	Locally adapt	http://link.	62	9	1635-1644	Brazil	South America	Authors one to six & e	Animal biometeor	Following exposu	ANOVA to compa
336	2020	Leite, Jacina	7	Coat color an	http://link.	64	12	2185-2194	Brazil	South America	Author one to two & fo	Animal biometeor	Environmental/m	Multivariate statist
337	2023	Li, Jie; Jia, Ku	5	Natural and sc	https://link	67	11	1789-1802	Brazil	South America	Authors one to five = (	Human biometeor	Data for this study	The Pearson corre
338	2022	Llop, María Je	5	Prediction of I	https://link	66	12	2529-2540	Argentina	South America	Authors one to five = A	Human biometeor	For three cities, w	Exploratory data a
339	2016	Locosselli, G	9	Rock outcrop	http://link.	60	5	639-649	Brazil	South America	Authors one to three =	Plant biometeorol	Data for this study	The authors applie
340	2017	Lucena, Reb	6	The Brazilian \	http://link.	61	12	2195-2203	Brazil	South America	Authors one to six = B	Human biometeor	Houly meteorolog	For each world cu
341	2014	DeSoto, Luci	7	Different grow	http://link.	58	10	2095-2109	Morocco; Spain	Africa; Europe	Authors one to seven	Plant biometeorol	The authors colle	To verify the CRU
342	2018	Lundgren-Ko	8	Climate chang	http://link.	62	3	347-358	India	Asia	Author one to three =	Human biometeor	The research tea	Descriptive statist
343	2018	Luo, Chuanx	10	Modern poller	http://link.	62	8	1471-1488	Northern Indian O	Asia	Authors one to nine =	Plant biometeorol	The authors colle	Abundance bar c
344	2015	Bradai, Lyès;	4	Effects of clim	http://link.	59	7	907-915	Algeria	Africa	Authors one to three =	Plant biometeorol	From seven sites	Descriptive statist
345	2022	Machado, Ní	9	Effect of dista	https://link	66	9	1853-1864	Brazil	South America	Authors one to two & f	Animal biometeor	Data (body weigh	The Kolmogorov r
346	2024	Machado, Th	6	Microclimate a	https://link	68	8	1519-1531	Brazil	South America	Authors one to six = B	Environmental bio	During 10 cloudle	Simple and mixed
347	2021	Macías-Cruz	7	n-6 Polyunsa	https://link	65	12	2077-2086	Mexico	South America	Authors one to seven	Animal biometeor	Data (weight, fee	A normality test (e
348	2016	Macías-Cruz	10	Effects of sea	http://link.	60	8	1279-1286	Mexico	South America	Authors one to ten = M	Animal biometeor	Data (blood meta	A MIXED procedu
349	2015	Maia, Paulo A	3	Wet-bulb glob	http://link.	59	10	1395-1403	Brazil	South America	Authors one to three =	Human biometeor	Data (air and dew	To assess the qua
350	2015	Maia, Alex Sa	6	Thermoregula	http://link.	59	8	1025-1033	Brazil	South America	Authors one to six = B	Animal biometeor	Data (height, bod	The least-square
351	2005	Campos Maia	3	Respiratory he	http://link.	49	5	332-336	Brazil	South America	Authors one to three =	Animal biometeor	Data (body surfac	Non-linear and m
352	2019	Maity, Anirud	6	Response of s	http://link.	63	2	143-152	India	Asia	Authors one to three,	Plant biometeorol	A two-year field e	ANOVA was appli
353	2012	Mejía-Domín	3	Spatial structu	http://link.	56	2	305-318	Mexico	South America	Authors one to three =	Plant biometeorol	Data (sapling hei	Kriging, a geostat
354	2020	Manavvi, S.; I	2	Semantics of	http://link.	64	2	253-264	India	Asia	Authors one & two = Ir	Human biometeor	During 5 summer	Descriptive statist
355	2015	Mandal, S.; C	3	Monsoon vari	http://link.	59	12	1891-1903	Sagar Island (Islan	Asia	Authors one to three =	Plant biometeorol	Meteorological da	Weekly rainfall pro
356	2021	Mandal, Dilip	3	Impact of envi	https://link	65	12	1999-2009	India	Asia	Authors one to three =	Animal biometeor	Meteorological da	Data were summa
357	2022	Mandal, Dilip	3	Effect of seas	https://link	66	11	2223-2235	India	Asia	Authors one to three =	Animal biometeor	The study used d	Duration of photop
358	2022	Manica, Ema	4	Changes in th	https://link	66	12	2477-2488	Brazil	South America	Authors one to four =	Animal biometeor	Daily data of max	Adequacy measu
359	2022	Marsh, Christ	7	Measuring an	https://link	66	6	1283-1295	Northern Sumatra	Asia	Authors one, two, four	Plant biometeorol	The authors colle	Descriptive statist
360	2010	Martello, Luc	4	Alternative bo	http://link.	54	6	647-652	Brazil	South America	Authors one to four =	Animal biometeor	Environmental dr	The effects of sea
361	2016	Martello, Luc	5	Infrared therm	http://link.	60	1	173-181	Brazil	South America	Brazil	Animal (cattle) bio	animal data, infra	Residual Feed Int
362	2017	Marwein, M. A	6	Response of v	http://link.	61	5	845-855	India	South Asia	India	Phenology, produ	Plant data, meas	Statistical analysis
363	2017	Marzouni, Mo	16	Health benefit	http://link.	61	8	1389-1401	Iran	Middle East	Iran	Air quality, human	Station data (PM	Macro coding in e
364	2022	Masroor, San	11	Effect of acut	https://link	66	9	1797-1809	India	South Asia	India	Animal (cattle) bio	Animal data, gen	Gene Expression
365	2020	Matthew, Ola	1	Investigating c	http://link.	64	3	355-365	Nigeria	West Africa	Nigeria	Climate, insects, d	rainfall, relative h	Statistical analysis
366	2014	Mendoza-He	7	Vegetation pa	http://link.	58	5	853-866	Mexico	North America	Mexico	Plant biometeorol	measured microc	Statistical analysis
367	2023	Mehta, Purni	2	Agromet whea	https://link	67	3	853–866	India	South Asia	India	Plant biometeorol	Measured tempe	Statistical analysis
368	2015	Menegassi, S	9	Scrotal infrare	http://link.	59	3	357-364	Brazil	South America	Brazil, USA	Animal biometeor	Animal data, Infra	Statistical analysis
369	2016	Menegassi, S	11	Effects of amb	http://link.	60	11	1787-1794	Brazil	South America	Brazil	Animal biometeor	Animal data, Infra	Statistical analysis
370	2016	Menegassi, S	11	The uses of in	http://link.	60	1	151-157	Brazil	South America	Brazil	Animal biometeor	Animal data, Infra	Statistical analysis
371	2024	Mir, Mohamm	5	Apple (Malus	https://link	68	5	927-938	India	South Asia	India	Plant biometeorol	Measured met. da	Statistical analysis
372	2022	Ali, Mohamm	4	Implications o	https://link	66	5	945-956	India	South Asia	India	Plant biometeorol	Measured met. da	Statistical analysis
373	2024	Molina, Julio	7	Bioclimatic th	https://link	68	2	253-261	Brazil	South America	Brazil, USA	Animal biometeor	Measured met. da	Statistical analysis
374	2021	Molina-Góme	5	Analysis of inc	https://link	65	1	119-132	Colombia	South America	Colombia, Spain	Air quality, human	Air quality monito	Exploratory analys
375	2016	Moradi Kor, N	3	The effects of	http://link.	60	5	757-762	Iran	Middle East	Iran	Animal biometeor	Site measuremen	Statistical analysis
376	2024	Moradpour, Z	5	Heat stress, th	https://link	68	12	2507-2514	Iran	Middle East	Iran	Human biometeor	Measured indices	Indices, question
377	2024	Muhammad,	4	Phleum prater	https://link	68	11	2227-2240	Pakistan	South Asia	Pakistan, USA	Plant biometeorol	Plant data, retriev	Statistical analysis
378	2022	Müller, Andre	6	Phenology of	https://link	66	11	2259-2271	Brazil	South America	Brazil	Plant biometeorol	Gridded met. and	Statistical analysis
379	2019	Nascimento,	6	Physiological	http://link.	63	11	1487-1496	Brazil	South America	Brazil, South Africa	Animal biometeor	Measured met. da	Principal Compon
380	2017	Ndetto, Emma	2	Assessment c	http://link.	61	1	69-85	Tanzania	East Africa	Tanzania, Germany	Human biometeor	Measured met. da	Statistical and Re
381	2021	Nedel, Ander	9	Analysis of inc	https://link	65	3	419-428	Brazil	South America	Brazil	Human biometeor	Measured indoor	Statistical analysis
382	2022	Neog, Prasar	5	Response of r	https://link	66	4	719-730	India	South Asia	India	Plant biometeorol	Met. station, plan	Statistical analysis
383	2022	Nick, Leticia	5	Relationship b	https://link	66	11	2301-2308	Brazil	South America	Brazil	Human biometeor	Daily Met. data (s	Statistical analysis
384	2021	Nili, Sairan; K	6	The effect of	https://link	65	11	1787-1797	Iran	Middle East	Iran, USA	Human biometeor	Cutaneous leishm	Statistical analysis
385	2017	Nouri, Milad;	3	Climate variab	http://link.	61	9	1571-1583	Iran	Middle East	Iran	Plant biometeorol	global climate va	Statistical analysis
386	2024	Nourmohamr	2	Benefits of ap	https://link	68	8	1573-1585	Iran	Middle East	Spain	Human biometeor	Hourly met. data,	Weather types, bi
387	2019	Nyssanbayev	5	Temporal dyn	http://link.	63	10	1381-1392	Kazakhstan	Central Asia	Kazakhstan, Spain	Human biometeor	Met. station, in-si	Statisctial analysis
388	2016	Aarif, Ovais;	2	Dry period coo	http://link.	60	3	465-473	India	Asia	India, India	Animal	Animal Data, met	Mixed effects mod
389	2024	Abbasi, Milad	5	Validity of ten	https://link	68	1	163-177	Iran	Asia	Iran, Iran, Iran, Iran, Ira	Thermal comfort a	meterological pa	Kolmogorov-Smir
390	2024	Abdalazeem	4	Impact of winc	https://link	68	7	1315-1326	Egypt	Africa and Asia	Egypt, Egypt, Egypt/Ja	Thermal comfort a	meterological pa	ENVI-met simulati
391	2001	el-Tayeb Abc	5	Effects of wall	http://link.	45	2	75-80	Sudan	Africa	Sudan, Sudan, Sudan	Plant productivity	Meterological pa	Comparison over t
392	2010	Adak, Tarun;	2	Quantifying th	http://link.	54	4	365-377	India	Asia	India, India	Plant productivity	Meterological pa	Linear regression
393	2022	Adegebo, Be	1	Urban therma	https://link	66	2	331-343	Nigeria	Africa	Nigeria	Human health	Meterological pa	multivariate analy
394	2016	El Yaacoubi,	7	Differentiated	http://link.	60	11	1695-1710	Morocco and Braz	Africa, South Ar	Morocco, Morocco, B	Plant productivity	Meterological pa	Bootstrapping to e
395	2020	El Yaacoubi,	8	Potential vuln	http://link.	64	3	377-387	Morocco	Africa	Morocco, Morocco, M	Plant productivity	Meterological pa	Partial least squar
396	2024	Afzal, Obaid;	6	CSM-CROPG	https://link	68	6	1213-1228	Pakistan	Asia	Pakistan, Pakistan, Pa	Plant productivity	Meterological pa	Generalized Likeli
397	2015	Aguilera, Fáti	10	Phenological	http://link.	59	5	629-641	Tunisia (Spain and	Africa	Italy, Italy, Italy, Italy, S	Plant productivity	Meterological pa	Least-squares reg
398	2017	Ahirwar, Man	10	Influence of s	http://link.	61	12	2119-2125	India	Asia	India, India, India, Indi	Animal	Animal Data, met	Generalised linea
399	2020	Ahmadi, Kou	6	The potential	https://link	64	9	1451-1462	Iran	Asia	Iran/Denmark, Iran, Ira	Plant productivity	Meterological pa	"Ten modeling alg
400	2020	Ajith, Y.; Dimr	10	Influence of w	https://link	64	10	1731-1742	India	Asia	India, India, India, Indi	Animal	Animal Data, met	Chi-Squared test

401	2014	Akbari, Mohs	2	Effects of diet	http://link.	58	6	1383-1391	Iran	Asia	Iran, Iran	Animal	Animal Data, met	Generalised linea
402	2016	Akbari, Mohs	3	Single and co	http://link.	60	3	447-454	Iran	Asia	Iran, Iran, Iran	Animal	Animal Data, met	General Linear Mc
403	2020	Akkemik, Üna	4	October to Ju	http://link.	64	5	803-813	Kazakhstan	Europe and Asi	Turkey, Turkey, Kazak	Climate	Meterological pa	Split sample proce
404	2018	Aleena, J.; S	6	Resilience of	http://link.	62	11	1995-2005	India	Asia	India, India, India, Indi	Animal	Animal Data, met	Shapiro-Wilk norm
405	2013	Alexander, P	1	Association o	http://link.	57	1	83-90	Argentina	South America	Argentina	Human Health	Human Health Da	t.test, Kolmogorov
406	2016	Alfonzo, Evel	8	Relationship b	http://link.	60	2	245-253	Brazil	South America	Brazil, Brazil, Brazil, Br	Animal	Animal Data, met	Analysis of Varian
407	2021	Alves, Mathe	10	Physiological	https://linl	65	12	2147-2155	Brazil	South America	Brazil, Brazil, Brazil, Br	Animal	Animal Data, met	Shapiro–Wilk norm
408	2024	Gupta, Aman	2	Enhancing th	https://linl	68	10	2083-2101	India	Asia	India, India	Urban and Building	Meterological dat	Segment mean sh
409	2020	Amjadian, Ta	2	Effects of repe	https://linl	64	12	2177-2183	Iran	Asia	Iran, Iran	Animal	Animal Data, met	ANOVA, generalis
410	2024	Anand, Shub	4	Comparative a	https://linl	68	9	1799-1810	India	Asia	India, India, India, Indi	Plant productivity a	Meterological pa	Machine learning
411	2014	Goswami, An	3	Effects of vita	http://link.	58	9	1961-1971	India	Asia	India, India, India	Animal	Animal Data	ANOVA, LSD pos
412	2023	Andrade, Ma	16	Determinants	https://linl	67	5	761-775	Brazil	South America	Brazil, Brazil, Brazil, Br	Animal	Animal Data, met	Hierarchical multip
413	2018	Aparecido, L	11	Neural networ	http://link.	62	11	1955-1962	Brazil	South America	Brazil, Brazil, Brazil, Br	Plant productivity a	Meterological dat	multilayer percept
414	2022	Araghi, Alirez	3	Effect of wind	https://linl	66	1	225-233	Iran	Asia	Iran, Iran, Denmark	Plant productivity a	Meterological pa	CERES-Wheat mc
415	2024	Araghi, Alirez	2	Evaluation of	https://linl	68	6	1201-1211	Iran	Asia	Iran, USA	Plant productivity a	Meterological pa	CERES-Wheat mc
416	2024	Arias, R. A.; K	4	Estimation of t	https://linl	68	9	1823-1836	Chile	South America	Chile, Chile, Chile, Chi	Animal biometeoro	Meteorological da	8 × 5 × 2 factorial
417	2024	Arora, Reena	9	Skeletal musc	https://linl	68	9	1811-1821	India	Asia	India, India, India, Indi	Animal biometeoro	Animal data (RNA	DAVID 6.8, CLC G
418	2022	Arsalani, Moh	3	Tree-ring-bas	https://linl	66	12	2553-2565	Iran	Asia	Germeny, Germeny, G	Plant biometeorolo	Plant data (earlyw	Regression mode
419	2024	Asad, Fayaz;	6	Tree-ring bas	https://linl	68	8	1545-1558	Pakistan	Asia	Pakistan, Pakistan, Pa	Plant biometeorolo	Plant data, meteo	Pearson correlatio
420	2024	Kc, Ashish; M	9	Development	https://linl	68	11	2205-2214	Nepal	Asia	Sweden, Nepal, Nepa	Health biometeoro	Human data (per	SPSS, bivariate c
421	2014	Athayde, Edu	2	Anthropogeni	http://link.	58	4	443-454	Brazil	South America	Brazil, Brazil	Plant biometeorolo	Plant data (speci	Tukey's honest st
422	2012	Avendaño-R	8	Physiological	http://link.	56	6	993-999	Mexico	North America	Mexico, Mexico, Mexi	Animal biometeoro	Animal data (rect	Thermal index (TH
423	2020	Babayan, L.	9	Influence of h	https://linl	64	12	2171-2176	Armenia	Asia	Armenia, Armenia, Arr	Health biometeoro	Human health da	atomic absorption
424	2023	Bajpai, Rajes	3	Unraveling the	https://linl	67	1	67-77	India	Asia	India, India, India	Plant biometeorolo	Plant data, meteo	Pigment analysis,
425	2020	Bal, Sourabh	2	Modeling and	https://linl	64	8	1379-1391	India	Asia	(Germany and India),	Animal /Health bio	Human health da	Autocorrelation ar
426	2022	Banerjee, Sh	3	A regression-	https://linl	66	2	313-329	India	Asia	India, USA, India	Health biometeoro	Human data (Que	MRT, ANCOVA wi
427	2011	Bannayan, M	5	Effects of pre	http://link.	55	3	387-401	Iran	Asia	Iran, Iran, Iran, Iran, Ira	Plant biometeorolo	Plant data (crop y	Climate indicies (A
428	2014	Bannayan, M	3	Estimating clir	http://link.	58	3	395-405	Iran	Asia	(Iran, USA), Iran, (Iran,	Plant biometeorolo	Meteorological da	Relative root mear
429	2020	Barreto, Cam	6	Infrared therm	http://link.	64	5	881-888	Brazil	South America	Brazil, Brazil, Brazil, Br	Plant biometeorolo	Plant data, meteo	Multiple factor ana
430	2018	Battisti, R.; S	3	Sensitivity and	http://link.	62	5	823-832	Brazil	South America	Brazil, Brazil, USA	Plant biometeorolo	Plant data, meteo	multi-model ense
431	2018	Berrama, Zah	6	The effects of	http://link.	62	6	1039-1048	Algeria	Africa	Algeria, Algeria, Alger	Animal biometeoro	Animal data, mete	statistical software
432	2017	Bhanuprakas	13	Comparative e	http://link.	61	5	931-941	India	Asia	India, India, India, Indi	Animal biometeoro	Animal data (bloc	SPSS (mean ± SE
433	2017	Bharati, Jaya	7	Expression dy	http://link.	61	8	1461-1469	India	Asia	India, India, India, Indi	Animal biometeoro	Animal data, mete	SPSS (mean ± SE
434	2014	Biudes, Marc	6	Ground and re	http://link.	58	6	1181-1193	Brazil	South America	Brazil, Brazil, Brazil, Br	Plant biometeorolo	Meteorological da	Willmott's index "c
435	2020	Bosco, L. C.;	3	Solar radiatior	https://linl	64	11	1969-1980	Brazil	South America	Brazil, Brazil, Brazil	Plant biometeorolo	Plant data (Leaf a	Tukey test, F test,
436	2020	Brand, Silvan	7	Effect of Alter	https://linl	64	12	2153-2160	Brazil	South America	Brazil, Brazil, Brazil, Br	Plant biometeorolo	Plant data (soil ch	Corrolation
437	2022	Brito, Amand	4	Heat wave ex	https://linl	66	12	2417-2424	Brazil	South America	Brazil, Brazil, Brazil, Br	Animal biometeoro	Animal data, mete	Thermal indices (T
438	2020	Bueno, João	8	Effect of cycli	http://link.	64	7	1125-1132	Brazil	South America	Brazil, Brazil, Brazil, Br	Animal biometeoro	Animal data, mete	Tukey test, Leven
439	2014	Burkart, Katri	6	An analysis of	http://link.	58	2	227-237	Bangladesh	Asia	Germany, Germany, G	Human health bior	Meteorological da	DLNM, Bayesian a
440	2016	Cabré, María	3	Regional clima	http://link.	60	9	1325-1340	Argentina	South America	Argentina, France, Ar	Climate and plant	Meteorological da	MM5 regional mod
441	2019	Campos, Firm	3	Outdoor envir	http://link.	63	11	1525-1532	Brazil	South America	Brazil, Brazil, Brazil	Human biometeor	Meteorological da	heat index, Stude
442	2019	Cardoso, Abr	7	How do metha	http://link.	63	11	1533-1540	Brazil	South America	Brazil, Brazil, Brazil, Br	Plant biometeorolo	Plant data (soil ch	Tukey test, one-w
443	2015	Cardozo, Nilc	5	Modeling suga	http://link.	59	12	1913-1925	Brazil	South America	Brazil, Brazil, Brazil, Br	Plant biometeorolo	Plant data, meteo	Regression analys
444	2018	Carvalho, Ra	7	Assessment c	http://link.	62	6	961-970	Brazil	South America	Brazil (Federal Univers	Plant biometeorolo	Meteorological da	Student's t test ar
445	2020	Casaroli, Derl	7	Transpiration	http://link.	64	1	1-13	Brazil	South America	Brazil	Plant	Plant data, meteo	Experimental desi
446	2013	Chakraborty,	2	A study on the	http://link.	57	5	769-774	India	Asia	India	Human (Health)	Physiological dat	ANOVA, Tukey's T
447	2014	Chand, Naila	5	Performance	http://link.	58	10	2153-2157	Pakistan	Asia	Pakistan (University of	Animal	Animal data, Phy	ANOVA, Duncan'
448	2017	Sarath Chand	8	Indian summe	http://link.	61	6	1063-1072	India	Asia	India (National Institut	Animal	Meteorological st	Descriptive statist
449	2014	Chen, Xiaoqi	5	Modeling gree	http://link.	58	4	463-471	Sri Lanka (City: Co	Asia	Sri Lanka (University c	Thermal Comfort a	Meteorological da	Simulation using F
450	2017	Chenchouni,	1	Variation in W	http://link.	61	3	549-564	Algeria	Africa	Algeria	Animal	Animal data, Mete	Generalized Linea
451	2022	Chikkagouda	9	Effect of heat	https://linl	66	5	995-1003	India	Asia	India, Australia, Bangl	Animal	Animal data, Mete	One-way ANOVA
452	2021	De Freitas, Cl	3	On-farm asse	https://linl	65	10	1659-1673	Brazil	South America	Brazil	Plant productivity a	Meteorological (s	Simulation modell
453	2012	Coelho, Mich	2	The impact of	http://link.	56	2	233-241	Brazil	South America	Brazil, United Kingdor	Human (Health)	Daily hospital adr	Negative Binomia
454	2021	Coelho, Ande	4	Optimization c	https://linl	65	11	1905-1917	Brazil	South America	Brazil (São Paulo Stat	Plant	Field experimenta	Polynomial regres
455	2014	De Melo Cost	5	Latent heat lo	http://link.	58	2	179-184	Brazil	South America	Brazil	Animal	Animal Data (eva	Mixed model analy
456	2010	Cueto, Rafae	3	Heat waves a	http://link.	54	4	335-345	Mexico	North America	Mexico (Universidad A	Thermal Comfort &	Meteorological (te	PET index modelir
457	2024	Da Conceiçã	6	Calibration, te	https://linl	68	9	1703-1716	Brazil	South America	Brazil (São Paulo Stat	Plant	Field experimenta	Root Mean Squar
458	2015	Da Costa, An	5	Rectal tempe	http://link.	59	11	1647-1653	Brazil	South America	Brazil	Animal	Quantitative phys	ANOVA (GLM pro
459	2010	Da Silva, Rob	3	Thermal radia	http://link.	54	1	5-11	Brazil	South America	All authors are affiliate	Animal	Meteorological (s	Empirical calculat
460	2019	Da Silva Ang	5	Arabica coffe	http://link.	63	7	831-843	Brazil	South America	Brazil	Plant	Phenological obs	ANOVA on ranks a
461	2024	Da Silva Dávi	4	The thermal c	https://linl	68	4	675-690	Brazil	South America	Brazil	Thermal Comfort &	Subjective survey	Pearson's chi-squ
462	2012	Da Silva, Rob	4	Latent heat lo	http://link.	56	5	927-932	Brazil	South America	Brazil	Animal	Empirical physiolo	Mixed models usir
463	2017	Da Silva, Wiln	8	Daily rhythmic	http://link.	61	7	1221-1231	Brazil	South America	Brazil; Spain	Animal	environmental va	Principal Compon
464	2015	Da Silva, Rob	3	Index of therm	http://link.	59	5	551-559	Brazil	South America	Brazil	Animal	Field observation	Principal Compon
465	2019	Da Silva, Igor	4	Excess of chil	http://link.	63	11	1517-1524	Brazil	South America	Brazil (Federal Univers	Human (Health)	Health data: Outp	Pearson correlatio
466	2021	Da Silva, Poll	6	An epidemiolo	http://link.	65	2	325-335	Brazil	South America	Brazil (Universidade F	Human (Health)	Climate data (Sta	Odds ratio with 95
467	2015	Da Costa, An	5	Hormonal prof	http://link.	59	2	231-236	Brazil	South America	Brazil (Universidade F	Animal	Primary field data	NOVA (SAS Proc
468	2017	Dadbakhsh,	4	Death from re	http://link.	61	2	239-246	Iran	Asia (Middle Eas	Iran & Australia	Human (Health)	Mortality records	Descriptive statist
469	2019	Dalla Costa,	5	How do seaso	http://link.	63	11	1497-1505	Brazil	South America	Brazil (Universidade E	Animal	Experimental anir	Factorial design (4
470	2014	Dangi, Satya	10	Impact of shor	http://link.	58	10	2085-2093	India	Asia	India (Indian Veterinar	Animal	Experimental anir	qRT-PCR, Wester
471	2015	Dangi, Satya	8	Expression of	http://link.	59	8	1095-1106	India	Asia	India (Indian Veterinar	Animal	Experimental phy	One-way ANOVA
472	2021	Sanagar Darl	4	Impacts of urb	https://linl	65	7	1101-1117	Iran	Asia (Middle Eas	Iran, Australia, Germa	Thermal Comfort &	Meteorological da	Microclimate simu
473	2013	Das, Tapasi;	2	Vegetative an	http://link.	57	1	185-196	India	Asia (South Asi	India (Department of E	Phenology	Phenological dat	Fortnightly and we
474	2018	Das, Bappa;	4	Evaluation of	http://link.	62	10	1809-1822	India	Asia	India	Agricultural	Rice yield time se	Stepwise multiple
475	2022	Das, Bappa;	4	Novel combin	https://linl	66	8	1627-1638	India	Asia	India	Agricultural	Daily weather dat	Stepwise linear re
476	2024	De Andrade I	10	Skin transcrip	https://linl	68	3	435-444	Brazil	South America	Brazil, U.S.	Animal	Experimental anir	Best unbiased line
477	2020	De Arêa Leão	3	Thermal sens	http://link.	64	3	533-545	Brazil	South America	Brazil	Human health	Survey data, hou	Linear regression
478	2020	De Castro Te	6	Modeling larg	https://linl	64	12	2053-2064	Brazil	South America	Brazil	Agricultural	Gridded climate c	Regression, algor
479	2017	De, Kalyan; K	5	Effect of high	http://link.	61	7	1269-1277	India	Asia	India	Animal	Experimental anir	General linear mo
480	2016	De Faria, Rog	5	Soybean yielc	http://link.	60	7	1015-1028	Brazil	South America	Brazil, U.S.	Agricultural	Weather data, ex	Mixed model meth
481	2022	De Farias Pa	3	Thermal sens	https://linl	66	3	469-480	Brazil	South America	Brazil	Human health	Survey data, hum	Canonical correla
482	2024	De França, L	8	Artificial neura	https://linl	68	11	2387-2398	Brazil	South America	Brazil	Agricultural	Experimental plar	Pearson correlatio
483	2003	De Garín, Alic	2	Mortality rate a	http://link.	48	1	31-36	Argentina	South America	Argentina	Human health	Weather/climate	Kolmogorov-Smir
484	2000	De Garín, A.;	5	Atmospheric c	http://link.	44	3	148-156	Argentina	South America	Argentina	Animal	Daily weather dat	Spearman and Ke
485	2003	De La Casa,	2	Assessing ten	http://link.	48	1	6-9	Argentina	South America	Argentina	Animal	Daily weather dat	Geospatial mappi
486	2017	De Mello, Juli	8	Physical and	http://link.	61	10	1723-1731	Brazil	South America	Brazil	Animal	Experimental anir	General linear mo
487	2022	De Oliveira A	7	Coffee pest se	https://linl	66	5	957-969	Brazil	South America	Brazil	Agricultural, anima	Climate data, ani	Pearson correlatio
488	2023	De Oliveira C	5	Thermal comf	https://linl	67	2	265-274	Brazil	South America	Brazil	Animal	Experimental anir	Kolmogorov–Smir
489	2023	De Oliveira, E	5	Maximum limit	https://linl	67	2	517-526	Brazil	South America	Brazil	Animal	Experimental anir	Pearson correlatio
490	2024	De Quadros,	5	Reliability of u	https://linl	68	12	2715-2729	Brazil	South America	Brazil, Italy	Human health	Weather data, we	Modeling
491	2014	De Queiroz, J	6	Daily variation	http://link.	58	6	1259-1264	Brazil	South America	Brazil	Animal	Experimental anir	General linear mo
492	2021	De Sousa, Ka	6	Developing of	https://linl	65	10	1781-1786	Brazil	South America	Brazil	Animal	Experimental anir	General linear mo
493	2022	De Souza E S	6	Thermal comf	https://linl	66	6	1079-1093	Brazil	South America	Brazil	Human health	Daily weather sta	Hotspot analysis
494	2014	De Souza, Pr	7	Physiological	http://link.	58	7	1559-1567	Brazil	South America	Brazil	Animal	Experimental anir	ANOVA, Pearson
495	2015	De Souza, Jo	5	The relationsh	http://link.	59	1	121-125	Brazil	South America	Brazil	Animal	Experimental anir	ANOVA, fixed effe
496	2016	De Souza Gr	3	PID temperatu	http://link.	60	8	1271-1277	Brazil	South America	Brazil	Animal	Experimental anir	ANOVA, descripti
497	2018	De Souza Gr	3	PID temperatu	http://link.	62	5	773-781	Brazil	South America	Brazil	Animal	Experimental anir	Kolmogorov-Smir
498	2020	De Vasconce	7	Adaptive profi	http://link.	64	1	105-113	Brazil	South America	Brazil	Animal	Weather data, an	Principal compon
499	2023	De-Sousa, K	5	Decision supp	https://linl	67	2	475-484	Brazil	South America	Brazil	Animal	Weather data, an	Fuzzy logic model
500	2015	Deb, Rajib; S	11	Understandin	http://link.	59	12	1783-1789	India	Asia	India	Animal	Animal data	ANOVA

501	2019	Deihimfard, R	3	Risk assessm	http://link.	63	4	511-521	Iran	Middle East	Iran, Australia	Agricultural	Daily weather dat	Modeling
502	2011	Del Ponte, Er	5	Early-season	http://link.	55	4	575-583	Brazil	South America	Brazil, U.S.	Agricultural	Rainfall data, sea	Empirical regressi
503	2019	Deniz, Mathe	5	High biodivers	http://link.	63	1	83-92	Brazil	South America	Brazil, U.S.	Agricultural, anima	Plant & weather d	Generalized linea
504	2020	Deniz, Mathe	6	Microclimate a	https://linl	64	11	1877-1887	Brazil	South America	Brazil, USA	Animal biometeoro	Microclimatic dat	Generalized Linea
505	2021	Dias, Henriqu	2	Assessing the	https://linl	65	11	1881-1893	Brazil	South America	Brazil	Agricultural	Daily gridded wea	Model simulations
506	2012	Dieng, Hama	8	The effects of	http://link.	56	1	113-120	Malaysia	Southeast Asia	Malaysia, Japan, Unit	Medical	Field and lab exp	Experimental man
507	2022	Do Nascimen	7	Thermal comf	https://linl	66	3	535-544	Brazil	South America	Brazil	Animal biometeoro	Environmental da	Field experiment c
508	2022	Dos Santos M	7	Microclimate a	https://linl	66	3	449-456	Brazil	South America	Brazil	Animal biometeoro	Environmental mi	Field measuremer
509	2024	Dutta, Gaura	8	Functional tra	https://linl	68	7	1371-1385	India	South Asia, Asia	India	Animal biometeorl	Whole transcripto	RNA-seq using st
510	2007	Duvail, Stéph	2	The Rufiji Rive	https://linl	52	1	33-42	Tazania	East Africa, Afri	Kenya, United Kingdo	Human biometeoro	Historical hydrolo	Mixed-methods a
511	2006	Johansson, E	2	The influence	http://link.	51	2	119-133	Sri Lanka	South Asia, Asia	Sweden, Sir Lanka	Urban	Meteorological fie	Field measuremer
512	2014	Falasca, Silvi	3	The agro-eco	http://link.	58	7	1433-1441	Argentina	South America	Argentina	Agricultural	Bioclimatic data -	Agroclimatic zonir
513	2015	Filali Ben Sid	8	Airborne fung	http://link.	59	3	339-346	Argentina	South America	Argentina	Environmental	Daily airborne spo	Descriptive statist
514	2022	Farahat, Ema	4	Wood anatom	https://linl	66	1	127-137	Egypt	North Africa, Afi	Egypt, Switzerland	Plant	Tree ring anatom	Wood anatomical
515	2022	Farias, Elizeu	5	Forecasting tl	https://linl	66	5	875-882	Brazil	South America	Brazil	Agricultural	Field-collected d	Artificial neural ne
516	2020	Faustini, Fab	3	The influence	http://link.	64	2	243-252	Brazil	South America	Brazil	Human biometeoro	Objective enviror	Field measuremer
517	2024	Ferag, Aziza;	7	Heat stress ef	https://linl	68	12	2515-2529	Algeria	North Africa, Afi	Algeria	Animal biometeorl	Animal reproduct	Statistical compai
518	2020	Ferreira, Nicc	2	Potential occu	http://link.	64	7	1051-1062	Brazil	South America	Brazil	Agricultural	Climate model pro	Geographic and t
519	2024	Ferreira, Josi	7	Assessment c	https://linl	68	7	1411-1419	Brazil	South America	Brazil	Animal biometeoro	Meteorological da	Comparative phys
520	2014	Fitchett, Jenr	4	Increasing fro	http://link.	58	8	1811-1815	Iran	Middle East, Asi	Iran	Agricultural	Historical meteor	Time series analys
521	2018	Fitchett, Jenr	2	Phenological	http://link.	62	6	1115-1119	Nigeria	West Africa, Afr	Nigeria	Agricultural	Qualitative data f	Purposive samplir
522	2017	Fonseca, Vir	8	Models to pre	http://link.	61	5	777-784	Brazil	South America	Brazil	Animal biometeoro	Experimental phy	Development of tw
523	2024	Fonsêca, V. I	11	Do newborn la	https://linl	68	9	1837-1846	Brazil	South America	Brazil	Animal biometeoro	Physiological dat	Field experiment v
524	2022	Freire, Anton	4	Analysis of the	https://linl	66	4	647-659	Brazil	South America	Brazil	Ecological	Micrometeorolog	Use of eddy covar
525	2020	Freitas, Aniel	6	Effect of therm	https://linl	64	10	1649-1656	Brazil	South America	Brazil	Animal biometeoro	Seminal quality d	Retrospective ana
526	2022	Gajjar, Hardik	3	Role of Azadir	https://linl	66	10	2055-2067	India	South Asia, Asia	India	Urban	Meteorological co	Field measuremer
527	2024	Gajjar, Hardik	4	Outdoor therm	https://linl	69	10	2871-2881	Brazil	South America	Brazil	Human biometeoro	Climate data - air	Field surveys with
528	2023	Garcia, Paulo	4	Thermal perfo	https://linl	67	10	1651-1658	Brazil	South America	Brazil	Animal biometeoro	Meteorological m	Field measuremer
529	2020	Garcia-Olive	13	Heat stress ef	https://linl	64	8	1367-1378	Brazil	South America	Brazil	Animal biometeoro	Lab experimenta	In vitro heat shock
530	2006	Gassmann, M	2	Trajectories a	http://link.	50	5	280-291	Argentina	South America	Argentina	Aerobiology	Daily airborne pol	Identification of po
531	2002	Gassmann, M	3	Sea-land bre	http://link.	46	3	118-125	Argentina	South America	Argentina	aerobiology, clima	Meteorological, a	Statistical analysis
532	2021	Gassmann, M	5	The impact of	https://linl	65	6	873-882	Argentina	South America	Argentina	Plant productivity	Meteorological, p	Destructive bioma
533	2019	Gautam, Priy	11	Inter-relations	http://link.	63	3	337-349	India	Asia	India	plant productivity	plant data, Meteo	Data analysis by u
534	2024	Gayari, Indraj	4	Elucidating th	https://linl	68	12	2641-2651	India	Asia	India	animal, thermal co	animal data, Mete	Statistical analyse
535	2020	Geirinhas, Jo	8	Heat-related	https://linl	64	8	1319-1332	Brazil	South America	Brazil, Portugal	thermal comfort &s	Meteorological, H	Negative binomial
536	2014	Ghasemi, Ho	3	Periodic usag	http://link.	58	7	1405-1414	Iran	Asia	Iran	animal, thermal co	animal data, Mete	Analysis of varian
537	2015	Ghazi, Shaha	3	Single and co	http://link.	59	8	1019-1024	Iran	Asia	Iran	animal, thermal co	animal data, Mete	Completely Rando
538	2017	Ghobadi, Mo	3	Effect of wate	http://link.	61	4	635-645	Iran	Asia	Iran	plant productivity &	plant data	Factorial Experime
539	2019	Ghosh, Satya	3	Evaluation of	http://link.	63	3	269-279	Kenya	Africa	India, UK	animal, thermal co	Meteorological, a	Thermal comfort a
540	2021	Ghosh, Satya	4	Engineered bi	https://linl	65	11	1967-1982	India	Asia	India, UK	urban & building, t	Meteorological, s	Satellite & GIS an
541	2017	Gill, Jaspreet	6	Cellular therm	http://link.	61	9	1687-1693	India	Asia	India	animal, thermal co	animal data	Descriptive statist
542	2018	Golher, Durg	9	Influence of s	http://link.	62	9	1709-1719	India	Asia	India	animal, climate	animal data, Mete	Descriptive statist
543	2018	Goudarzi, Gh	12	Association b	https://linl	62	8	1461-1470	Iran	Asia	Iran	human health, clin	human health, er	Descriptive statist
544	2023	Guesine, Gio	3	Thermoregula	https://linl	67	6	1003-1015	Brazil	South America	Brazil	animal, thermal co	Meteorological, a	Descriptive statist
545	2023	Guesine, Gio	3	Spatial model	https://linl	67	11	1775-1788	Brazil	South America	Brazil	animal, thermal co	microclimatic dat	Descriptive statist
546	2022	Gujar, Gayatı	8	Characterizat	https://linl	66	8	1515-1529	India	Asia	India	animal, thermal co	Meteorological, a	One-way ANOVA
547	2020	Karnatak, Gu	9	Modeling pre-	https://linl	64	11	1889-1898	India	Asia	India, Czech Republic	animal, thermal co	animal data, Mete	Descriptive statist
548	2019	Guo, Wenfan	15	The effect cha	http://link.	63	2	159-166	Mongolia	Asia	China	thermal comfort &s	Meteorological, H	Descriptive statist
549	2021	Gutierrez, J. I	1	Effects of met	https://linl	65	2	257-263	Colombia	South America	South America	human health, clin	Meteorological, H	Multilevel negative
550	2024	Havenga, H.;	12	Healthy enviro	https://linl	68	9	1757-1771	South Africa	Africa	South Africa	human health, aer	Meteorological, H	Descriptive botan
551	2023	Hachisuca, A	6	Growing degr	https://linl	67	7	1169-1183	Brazil	South America	Brazil	phenology, plant p	Meteorological, p	Descriptive Statis
552	2016	Haque, N.; Si	3	Up-regulation	http://link.	60	12	1819-1828	India	Asia	India	animal, thermal co	Meteorological, a	Descriptive statist
553	2023	Hassan, Sab	4	Blood hemato	https://linl	67	4	675-686	Egypt	Africa and Asia	Egypt	animal, thermal co	Meteorological, a	general linear mod
554	2015	Hassanpour,	5	Oxidant and e	http://link.	59	11	1615-1621	Iran	Asia	Iran	animal, bird	animal data	Descriptive Statis
555	2024	Hemanth, M.:	7	Comparative a	https://linl	68	11	2267-2279	India	Asia	India	animal, bird, therm	Meteorological, a	Descriptive Statis
556	2016	Hofmann, Ga	5	Implications o	http://link.	60	3	421-433	Brazil	South America	Brazil	animal, thermal co	Meteorological, a	Circular Statistics.
557	2018	Holcman, Est	4	Vineyard micr	http://link.	62	6	925-937	Brazil	South America	Brazil	plant productivity &	Meteorological, p	Descriptive statist
558	2018	Hooper, Heni	5	Acute heat st	http://link.	62	12	2257-2265	Brazil	South America	Brazil	animal, bird, therm	Meteorological, a	Shapiro-Wilk Test
559	2019	Hooper, Heni	12	Heat loss effic	http://link.	63	11	1475-1486	Brazil	South America	Brazil, Portugal	animal, thermal co	Meteorological, a	F test and Studen
560	2021	Hooper, Heni	6	Long-term he	https://linl	65	12	2123-2135	Brazil	South America	Brazil	animal, thermal co	Meteorological, a	Descriptive Statis
561	2024	Hunt, Andrew	10	Evidence of h	https://linl	68	6	1169-1178	247 people compl	South America	Brazil	Thermal comfort, h	Qualitative data f	Distribution via a S
562	2017	Ihsanullah; Q	5	Postpartum e	http://link.	61	9	1561-1569	Pakistan	Asia, South Asia	Pakistan	Animal heat stress	Animal and met-c	Analysis of varian
563	2024	Raissouni, Ijla	4	Variations, tre	https://linl	68	12	2613-2625	Morocco	Africa	Morocco, Spain	plant productivity,	Aerobiology and	The Kolmogorov-
564	2018	Ikefuti, Priscil	3	Mean air temp	http://link.	62	8	1535-1542	Brazil	South Americar	Brazil	urban climate, the	human health an	Quasi-Poisson re
565	2017	Ingole, Vijenc	7	Socioenviron	http://link.	61	10	1797-1804	India	Asia, South Asia	India, Sweden, Ghana	Thermal comfort	Human health an	quasi-Poisson reg
566	2016	Faseleh Jahr	8	Dietary supple	http://link.	60	7	1099-1110	Malaysia	Asia	Malaysia, Italy	Animal thermal co	Animal, physiolog	one-way ANOVA
567	2024	JawaharJoth	7	Effect of tropo	https://linl	68	5	991-1004	India	Asia, South Asia	India	Agriculture, met-d	Plant physiologic	SSPS (version 16)
568	2016	Jayakumar, M	3	Climate-base	http://link.	60	12	1943-1952	India	Asia, South Asia	India	Plant productivity	Agro-met data	t test
569	2021	Jayasinghe,	3	The future of	https://linl	65	10	1629-1646	Sri Lanka	Asia, South Asia	Australia, Sri Lanka	Climate, agricultur	climate, plant, ag	three-way ANOVA
570	2024	Jayasinghe,	4	Effects of stre	https://linl	68	6	1133-1142	Sri Lanka	Asia, South Asia	Canada	Urban & building,	Microclimate, veg	ENVI-met simulati
571	2018	Johansson, E	4	Outdoor therm	http://link.	62	3	387-399	Ecuador	South America	Sweden, Ecuador, Fir	human thermal str	human, climate, u	questionnaire sur
572	2017	Kahwage, Pr	10	High systemic	http://link.	61	10	1819-1829	Brazil	South America	Brazil	Animal and climate	animal, climate, b	analysis of varian
573	2022	Kalyankumar	7	Molecular epi	https://linl	66	10	1985-1995	India	Asia, South Asia	India	Plant productivity	plant, climate, en	R studio
574	2022	Kappes, Rob	8	Rumination tir	https://linl	66	4	791-801	Brazil	South America	Brazil	Animal thermal co	Animal behavior,	physiological, proc
575	2024	Karuppanna	7	Forecasting tl	https://linl	68	9	1871-1884	India	Asia, South Asia	India, Kenya	pest and crop eco	Biodiversity, ecol	Species Distributi
576	2016	Karvatte, Niv	6	Shading effec	http://link.	60	12	1933-1941	Brazil	South America	Brazil	livestock thermal c	climate, soil, vege	ANOVA
577	2024	Kashyap, Gir	7	Machine learr	https://linl	68	6	1179-1197	Brazil	South America	Brazil	crop-livestock-for	agriculture, fores	ANOVA/MANOVA
578	2015	Khalid, Bushi	2	Dengue trans	http://link.	59	3	267-283	Pakistan	Asia, South Asia	Pakistan	Human health wea	Entromology, hur	Multilevel modelin
579	2016	Khan, Amin U	3	Establishing a	http://link.	60	12	1897-1906	Pakistan	Asia, South Asia	Pakistan	Plant productivity	Species occurre	MaxEnt
580	2015	Khosravinia,	1	Effects of Sat	http://link.	59	11	1711-1719	Iran	Asia, South Asia	Iran	Animal, thermal co	Animal health and	SkEO i - model
581	2018	Krishnan, G.;	6	Adaptation st	http://link.	62	8	1497-1506	India	Asia, South Asia	India	Animal comfort an	animal behaviour	ANOVA
582	2017	Krüger, E; Dra	3	Outdoor comf	http://link.	61	3	463-475	Brazil	South America	Brazil, Germany	Thermal comfort &	Surveys/interview	Regression analys
583	2017	Krüger, E; Ro	3	Calibration of	http://link.	61	7	1323-1336	Brazil	South America	Brazil	human biometeoro	Human physiolog	PET index calibrat
584	2019	Krüger, E. L.;	4	Identifying sol	http://link.	63	3	301-313	Brazil	South America	Brazil	thermal comfort &	Behavioral mapp	Space syntax ana
585	2021	Krüger, Edua	5	Calibrating UT	https://linl	65	9	1463-1472	Brazil	South America	Brazil	human biometeoro	Human thermal p	UTCI recalibration
586	2023	Krüger, Edua	4	Analyzing the	https://linl	67	9	1461-1475	Brazil	South America	Brazil	climate-health inte	health and met da	Distributed lag nor
587	2024	Krüger, Edua	4	Introducing PI	https://linl	68	11	2357-2371	Brazil	South America	Brazil	human-environme	Microclimate mea	Correlation analys
588	2022	Kumar, Navn	2	Interaction of	https://linl	66	7	1473-1485	India	Asia, South Asia	India	aerobiology; clima	Aerosol, met-dat	Principal Compon
589	2016	Kumar, Anil; \	8	Assessment c	http://link.	60	6	873-882	India	Asia, South Asia	India	cattle thermal ada	Animal, climate a	Micronucleus test
590	2023	Kumar, P. Vij	3	Spatial and te	https://linl	67	3	485-501	India	Asia, South Asia	India	crop-climate relati	Agrometeorologi	Mann-Kendall tes
591	2024	Kumar, Prabl	3	Effect of short	https://linl	68	4	795-806	India	Asia, South Asia	India	goat adaptation pl	Animal, breed an	Mixed-effects mo

2) List of Global South countries with an identifying reference.

Country	Reference to Support inclusion (just one)	Number of papers found in IJBM 2000-2024
Afghanistan	https://doi.org/10.1177/0042098013487773	14
Albania		x
Algeria	https://doi.org/10.1093/migration/mnz035	33
* American Samoa	Kowalski, A.M. (2020) Global South-Global North Differences.	1
Angola	https://doi.org/10.1111/geoj.12334	13
Anguilla		x
Antigua + Barbuda	https://link.springer.com/chapter/10.1007/978-3-030-24788-1_6	0
Argentina	https://doi.org/10.1016/j.ecolecon.2023.107946	82
Armenia	http://dx.doi.org/10.1080/01436597.2015.1128817	4
Aruba	https://www.researchgate.net/publication/277021215_An_Expl	0
Azerbaijan	https://doi.org/10.1080/01436597.2025.2482992	8
Bahamas	https://doi.org/10.1016/S0025-326X(00)00104-1	1
Bangladesh	https://doi.org/10.1016/j.uclim.2024.102072	36
Barbados	https://www.researchgate.net/publication/373482377_Climate_	14
Belarus	In Global inequality literature it is usually placed in the Global I	x
Belize	https://onlinelibrary.wiley.com/doi/full/10.1111/j.1468-2427.200	3
Benin	https://www.academia.edu/download/122661752/Land_Grabbir	7
Bermuda	Not found to be Global South. British overseas territory and ma	x
Bhutan	https://www.mdpi.com/2071-1050/17/7/3261	9
Bolivia	https://www.pseau.org/outils/ouvrages/researchgate_participat	10
Bosnia & Herzegovina	Does not fit the traditional definitions of the Global North/South	x
Bostwana	https://doi.org/10.1080/23311886.2025.2480726	11
Brazil	https://doi.org/10.1080/08039410.2025.2467651	336
British Virgin Islands (BVI)	https://doi.org/10.1007/978-3-030-80791-7_9	x
Brunei	Kowalski, A.M. (2020) Global South-Global North Differences.	3
Bulgaria	https://doi.org/10.1080/14650045.2018.1477757	x
Burkina Faso	https://doi.org/10.1080/23311886.2024.2321710	12
Burundi	https://doi.org/10.1007/s12571-024-01482-9	4
Cabo Verde	https://doi.org/10.1002/aqc.4045	0
Cambodia	https://doi.org/10.1016/j.ijinfomgt.2021.102454	11
Cameroon	https://doi.org/10.1016/j.jenvman.2019.03.098	10
Cayman Island	https://doi.org/10.1007/978-3-319-95714-2_68	0
Central Africa Republic	https://doi.org/10.1007/978-3-319-95714-2_68	21
Chad	https://doi.org/10.3390/urbansci9020022	7
Chile	https://doi.org/10.1016/j.resconrec.2019.104441	48
Cocos (Iceeling) Islandi		x
Colombia	https://link.springer.com/chapter/10.1057/978-1-137-53969-4_1	27
Comoros	Kowalski, A.M. (2020) Global South-Global North Differences.	0
Cook Islands	No direct global south reference	x
DRC	https://doi.org/10.1080/14747731.2024.2374432	3
Republic of Congo	https://doi.org/10.1080/03056244.2023.2277616	6
Costa Rica	https://doi.org/10.1016/j.scitotenv.2024.173396	13
Côte d'Ivoire	https://www.idos-research.de/fileadmin/migratedNewsAssets/F	0
Cuba	https://doi.org/10.1111/imig.12127	20
Curaçao	Kowalski, A.M. (2020) Global South-Global North Differences.	0
Cyprus	https://doi.org/10.1080/02589001.2023.2195601	22
Djibouti	https://doi.org/10.1080/17531055.2024.2512640	1
Dominica	Kowalski, A.M. (2020) Global South-Global North Differences.	0
Dominican Republic	https://doi.org/10.1525/sod.2025.2478514	2
Timor-Leste	https://link.springer.com/article/10.1007/s10993-020-09570-0	1
Ecuador	https://onlinelibrary.wiley.com/doi/10.1111/j.1467-8330.2007.0	17
Egypt	https://doi.org/10.1093/jrs/fel014	37
El Salvador	https://onlinelibrary.wiley.com/doi/10.1111/anti.13137	2
Equitorial Guinea	https://www.cambridge.org/core/journals/africa/article/abs/extra	1
Eritrea	https://doi.org/10.1177/22779760241264433	1
Eswatini	https://doi.org/10.2989/16085906.2023.2270963	5
Ethiopia	Odeh, 2010 "A comparative analysis of global north and global	25
Fiji	Fraenkel, J. (2024). The Pacific Islands: The Centrality of Cont	7
French Guiana	Bishop, M. L., Clegg, P., & Hoefte, R. (2016). Hemispheric reg	6
French Polynesia	Fraenkel, J. (2024). The Pacific Islands: The Centrality of Cont	3
Gabon	Ifesinachi Okafor-Yarwood & Ibukun Jacob Adewumi (2020) To	3
The Gambia	https://www.jstor.org/stable/48767451	7
*Georgia	https://doi.org/10.1080/1060586X.2023.2289309	x
Ghana	https://doi.org/10.1016/j.rser.2020.109830	19

*Greece	https://doi.org/10.1386/jgmc_00054_2		
Grenada	https://doi.org/10.1007/978-3-031-30889-5_2		1
Guadeloupe	https://doi.org/10.1080/2833115X.2024.2398511	x	
*Guam	Kowalski, A.M. (2020) Global South-Global North Differences.		2
Guatemala	https://doi.org/10.1080/09687599.2016.1214425		6
Guinea	https://doi.org/10.1080/00020184.2020.1827947		49
Guinea-Bissau	Martins, A. and Oliveira Moreira, C., 2024. Tourism or sustaina		1
Guyana	Obeng-Odoom, F., 2025. Developmental Environmentalism in (		6
Haiti	Pickup, M., 2018. Evaluating brazilian south–south cooperatio		2
Honduras	Shipley, T., 2016. Enclosing the commons in Honduras. Amer		3
India	Gerber, J.F. and Raina, R.S., 2018. Post-growth in the global s		286
Indonesia	Zain, A.F., Pribadi, D.O. and Indraprahasta, G.S., 2022. Revisi		40
Iran	Vahabli, D., 2025. From the global South to the human rights s		134
Iraq	https://ijbes.utm.my/index.php/ijbes/article/view/871		7
Jamaica	https://books.google.co.za/books?hl=en&lr=&id=5ySMchaCjC		2
Jordan	https://academic.oup.com/jrs/article-abstract/37/2/589/761963		38
Kazakstan	https://www.tandfonline.com/doi/full/10.1080/14767724.2025.2		0
Kenya	https://www.mdpi.com/1996-1073/14/14/4362		27
Kiribata	https://www.ssoar.info/ssoar/handle/document/57809		0
Kosovo	https://www.taylorfrancis.com/chapters/edit/10.4324/97804295		3
Kyrgyzstan	Kowalski, A.M. (2020) Global South-Global North Differences.		5
Laos	Phaahla, E. (2024). The Political Economy of Divergent Welfar		8
Lebanon	Kowalski, A.M. (2020) Global South-Global North Differences.		3
Lesotho	Kowalski, A.M. (2020) Global South-Global North Differences.		2
Liberia	Kowalski, A.M. (2020) Global South-Global North Differences.		5
Libya	Kowalski, A.M. (2020) Global South-Global North Differences.		6
Madagascar	Kowalski, A.M. (2020) Global South-Global North Differences.		12
Malaysia	https://doi.org/10.1177/0042098018812009		44
Malawi	https://doi.org/10.1186/s12910-023-00889-x		3
Maldives	https://doi.org/10.24043/isj.177 / Karthikheyan, T. C. 2010. En		0
Mali	https://doi.org/10.1080/01436597.2021.1965870		8
Marshall Islands	https://doi.org/10.1017/S0922156517000401		1
Martinique	https://doi.org/10.4000/etudescaribeennes.6073 / Marques, B.	x	
Mauritania	Kowalski, A.M. (2020). Global South-Global North Differences.		10
Mauritius	http://dx.doi.org/10.1016/j.apgeog.2016.12.014		3
Mayotte	Mori, M., Longépée, E., Lefer-Sauvage, G., Banos, A., Becu, N		1
Mexico	Quiroga-Garza, A., Garza-Cisneros, A. N., Elizondo-Omaña, R		129
Micronesia	https://www.bisa.ac.uk/become-a-member/global-south-countr		2
Moldova	Lupușor, A., CENUSA, D., MORCOTILO, I., & PROHNITCHI, V		8
Mongolia	https://www.bisa.ac.uk/become-a-member/global-south-countr		58
Montenegro	https://worldpopulationreview.com/country-rankings/global-sout	x	
Montserrat	No reference		x
Morocco	Campitelli, A., Aryoug, O., Ouazzani, N., Bockreis, A. and Scl		32
Mozambique	Arsanjani, J.J., Fibæk, C.S. and Vaz, E., 2018. Development c		10
Myanmar	Thinh, M.P., 2025. Rethinking educational equity in the Global		12
Namibia	Kimaro, M.E. and Saarinen, J., 2019. Tourism and poverty alle		10
Nauru	Morris, J., 2021. Colonial afterlives of infrastructure: from phosp		0
Nepal	Aryal, K., Laudari, H.K., Neupane, P.R. and Maraseni, T., 202		26
Netherlands Antilles	no reference		x
New Caledonia	Batterbury, S.P., Kowasch, M. and Bouard, S., 2020. The geo		3
Nicaragua	Alvarado, N.A., 2022. Migrant politics in the urban Global Sout		8
Niger	E. Ite, U., 2004. Multinationals and corporate social responsibi		19
Nigeria	Nwosu, C.F. and Vogt, M., 2024. A Cultural Approach to Posto		50
Niue	Implied>>Watson, R. and Nel, E., 2020. Applying developmen		x
North Korea	Kowalski, A.M., 2020. Global south-global north differences. In		1

North Macedonia	Disputed>>Abduli, S., Arifi, A., Ibraimi, S. and Zuferi, R., 2022	x
Northern Mariana Islands	Kowalski, A.M., 2020. Global south-global north differences. In	0
Pakistan	Shakil, K. and Yilmaz, I., 2021. Religion and populism in the G	63
Palau	Harwit, E., 2000. Taiwan's foreign economic relations with deve	1
Palestine	Safadi, Najwa Sado (07/2011). "Exploring the Relationship Bet	0
Panama	Mollett, S. (2023). Racial Geographies of Land and Domestic S	8
Papau New Guinea	Vigya Sharma, Anthony P. Heynen, Nicholas Bainton, John Bu	0
Paraguay	Ioris, A.A.R. (2024), 'Development, subtraction and the Indigen	12
Peru	Stéphanie Rousseau , Social Politics: International Studies in	24
Philippines	Misalucha, C.G. The challenges facing the global south: persp	23
Pitcairn Islands	No reference	x
Puerto Rico	Kowalski, A.M., 2020. Global south-global north differences. In	15
Romania	Not considered part of the Global South/No reference	x
Rwanda	Dufitimana, E., Gahungu, P., Uwayezu, E., Mugisha, E., Poor	8
St. Helena	No reference	x
St Kitts & Nevis	Corbett, J., & Byron, J. (2023). Secessionism in Nevis: Why H	0
St Lucia	A. Wahab (2018) 'Queer affirmations: negotiating the possibilit	6
St Vincent & Grenadines	Braithwaite, B., 2020. Ideologies of linguistic research on smal	x
Samoa	Thornton, A., Kerslake, M.T. and Binns, T., 2010. Alienation ar	1
Senegal	Heng, S., Tsilionis, K., Scharff, C. and Wautelet, Y., 2022. Un	7
Serbia	Vidojević, J. and Nakarada, R., 2023. Basic Analytical Assum	43
Seychelles	https://www.researchgate.net/publication/352126836_Global_S	1
Sieria Leone	https://www.researchgate.net/publication/352126836_Global_S	5
St Maarten	Identified as global North	x
Solomon Islands	https://www.researchgate.net/publication/352126836_Global_S	1
Somalia	https://www.researchgate.net/publication/352126836_Global_S	6
South Africa	https://www.researchgate.net/publication/352126836_Global_S	72
Sri Lanka	https://link.springer.com/article/10.1007/s10639-019-10069-3	35
South Sudan	Kowalski, A.M. (2020) Global South-Global North Differences.	4
Sudan	Kowalski, A.M. (2020) Global South-Global North Differences.	18
Suriname	Kowalski, A.M. (2020) Global South-Global North Differences.	5
Syria (Syrian Arab Republic)	Kowalski, A.M. (2020) Global South-Global North Differences.	15
Tajikistan	Kowalski, A.M. (2020) Global South-Global North Differences.	3
Tanzania	https://www.tandfonline.com/doi/full/10.1080/25729861.2021.1	33
Ukraine	No Global South reference	x
*US Virgin Islands	Kowalski, A.M. (2020) Global South-Global North Differences.	1
Urugauy	https://link.springer.com/chapter/10.1007/978-3-319-62554-6_7	17
Uzbekistan	https://www.tandfonline.com/doi/full/10.1080/13563467.2023.2	7
Vanuatu	Kowalski, A.M. (2020) Global South-Global North Differences.	1
Venezuela	https://journals.sagepub.com/doi/full/10.1177/0263395721106	8
Vietnam	https://doi.org/10.1177/1461444816629475	40
*Wallis & Futuna	No scientific references	x
Western Sahara	https://doi.org/10.1080/13629387.2025.2500472	5
Yemen	https://doi.org/10.1177/0306396818812001	1
Zambia	https://doi.org/10.1080/23792949.2016.1188665	17
Zimbabwe	https://doi.org/10.1007/978-3-030-92114-9_4#DOI	53
Thailand	https://www.tandfonline.com/doi/abs/10.1080/19460171.2017.1	10
Togo	Kowalski, A.M. (2020) Global South-Global North Differences.	4
Trinidad Tobago	https://www.taylorfrancis.com/chapters/edit/10.4324/97810031	22
Tunisia	https://www.ssoar.info/ssoar/handle/document/49030	4
Turkmenistan	Kowalski, A.M. (2020) Global South-Global North Differences.	0
Tuvalu	chrome-extension://efaidnbmnnnibpcajpcglclefindmkaj/https://e	0
Turks and Caicos	Kowalski, A.M. (2020) Global South-Global North Differences.	9
Uganda	https://journals.sagepub.com/doi/full/10.1177/14730952221076	1

3) VOS viewer showing co-citation between Global South publications.

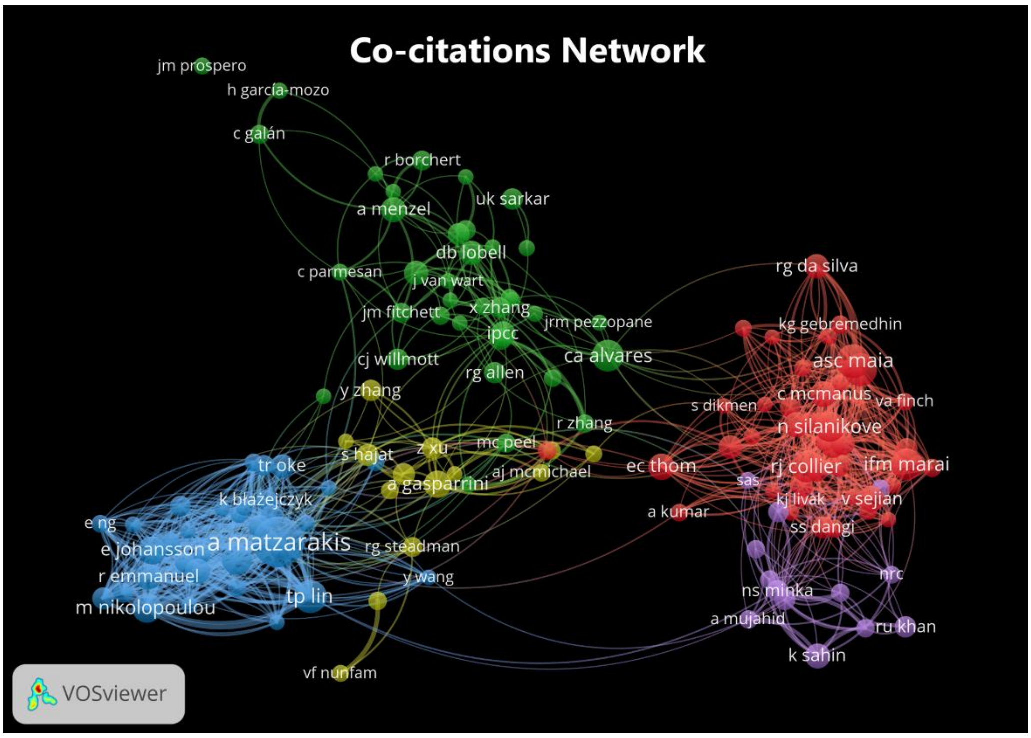

4) Positionality statements of the 26 authors involved in this study.

Ariel Prinsloo: As a middle-class white female with a PhD-level education, born and raised in South Africa, I have been exposed to a diversity of cultures and socio-economic circumstances. Within the context of South Africa, I acknowledge my privilege through my access to educational resources as well as financial opportunities that have allowed me to travel to the United States of America, Italy, Ireland, Réunion Island and areas of southern Africa such as Lesotho, Mozambique and Zimbabwe. These have impacted my positionality lens.

Claire Gallacher: I acknowledge my position as a highly educated white female researcher with a BSc in geography, an MSc in environmental governance, and a PhD in environmental science. Although I was raised in Scotland, I have spent the majority of my adult life in Germany, alongside extended research and academic stays in Malaysia, Trinidad & Tobago and New York. These diverse experiences and my educational background add nuance to my understanding of the Global South, but I remain aware that my background in the Global North limits my perspective. I am committed to engaging with the local knowledge and experiences from the coauthors from countries in the Global South, being mindful of the limitations of my own background, and ensuring that my research respects the diversity and complexity of the Global South's environmental and socio-political realities.

Felix Adebayo: I am a Black male of African descent from the Southwestern region of Nigeria. Growing up, formal education was not widely emphasized, which fueled my determination to pursue it and inspired many children in the community. Thanks to my parents' support and my own determination, I persisted in pursuing education despite these challenges, aiming to make society a better place through learning and contributing solutions to pressing environmental issues. I hold a BSc in Meteorology and a Master's in GIS and Remote Sensing, and I am currently a PhD student at the University of Waterloo, Canada. Having lived and studied in both the Global South and the Global North, I am strongly aware of how environmental risks and policy responses affect communities differently, depending on their social and economic contexts.

Betty Adegebo: I am a black, straight female Health Geographer/Bioclimatologist, who was born and brought up in the southwestern part of Nigeria. My positionality is shaped by my lived experiences and education in a country that continues to face the legacies of colonialism. As an early career researcher (a year post Ph.D.), I am focused on building my professional identity in the context of urban climate, human and environmental health. I am also committed to converting research findings into public knowledge through climate and health literacy programs/ platforms and environment-related policy advocacy particularly in vulnerable communities. I acknowledge the limitations my identity, society, educational and cultural background place on my research and career development particularly in a field (Climatology) dominated by men. I have had the academic privilege of visiting diverse institutions across both global north and global south, including South Africa, Germany, Canada, Ghana, and Australia. I am grateful to the funding bodies and institutional access to educational resources that enabled these experiences, allowing me to explore various constructs of human societies, climate disparities, and cultural dimensions. These lived experiences and academic stays shaped my understanding of "The Global South".

Adnan Arshad: As a South Asian male from a rural Punjabi farming community in Pakistan, my background profoundly shapes my research on Agricultural Meteorology. Being the first in my family to attend a university, I recognize education's transformative power while remaining critical of systemic inequities. My journey from a village school to a PhD in China fuels my commitment to inclusive, need-based research that centers on farmers, especially those vulnerable to changing climatic conditions. My identity informs my focus on participatory, locally grounded solutions, though I acknowledge my privileges as an agrometeorologist. Working across Pakistan and China, I strive to bridge global climate agriculture science and community needs while challenging power imbalances in knowledge production, research translation, and transformation for informed farmer communities. When to grow, how to grow, and how much to produce by linking meteorology services into agriculture.

Shreya Banerjee: I am a brown straight female born and raised in India, having a Master's and a PhD degree from IIT Kharagpur, India- an institute with .01% acceptance rate. I am a third-generation engineer in my family, and as a South Asian female, I am aware of my privileges in terms of education and access to opportunities. I grew up in a small coal mining town in Eastern India in an affluent, secular and highly educated family with a wide exposure of world literature, global politics and current affairs. At the same time, I witnessed significant inequality in my surroundings through my everyday interaction with people from various social strata. Over the years, I have travelled extensively across India, Southeast Asia, Africa, Australia, the United States, and Europe for my leisure, work and research stays, enabling me to recognize the contrast between the Global North and the Global South prominently. Currently, as a female academic in a resource-constrained region in Western India, my everyday experiences of gender inequalities, socio-cultural stigmas, and inadequate development level also shape my positionality. I am committed to bridging the north-south gap

by contributing meaningfully with my research to the society by acknowledging the requirements of various stakeholders. Empathy plays an important role in how I see the world.

Elizabeth Carr: I am a white, English-speaking female who grew up in a middle-class family in a small, predominantly Afrikaans farming town in the Mpumalanga province of South Africa. Both my parents attained tertiary education and I have similarly pursued higher education. Most of my schooling and academic training occurred in racially, culturally and politically diverse environments. My academic background lies in Geography, encompassing both physical and human geography, which has shaped my understanding of environmental and social dynamics.

While I have not yet travelled outside of Africa, I have explored South Africa extensively for leisure and academic purposes. I have also visited the Kingdom of Lesotho, the Kingdom of Eswatini and the Republic of Mauritius. These experiences have broadened my perspective on the Global South and the challenges faced.

Although I grew up in a Global South country and have personally encountered some challenges often associated with this context, I acknowledge that my experiences are mediated by my racial identity, socio-economic status and urban upbringing. As a white individual from a middle-income background, I recognize that my worldview and interpretations differ significantly from those of people of colour, as well as individuals from lower-income or rural communities within the Global South. Having grown up in post-apartheid South Africa, with parents who experienced life during apartheid, I have grown up acutely aware of the legacies of systemic inequality and the ways these continue to shape identities and perspectives today. I am conscious of the privileges I hold and the limitations they may impose on my ability to fully represent or understand the lived experiences of more marginalized populations.

Thea Juanita Earnest: As a first year Master of Science (MSc) student pursuing my degree at the University of the Witwatersrand in Health Biometeorology, my positionality is shaped by my lived experience as a heterosexual Indian female that was raised by a middle class family in a small town on the east coast of South Africa, my exposure to varying cultural and socio-economic contexts through experience and access to opportunity, as well as my education. Both my parents grew up in Apartheid South Africa and did not have access to financial resources to pursue degrees at a tertiary institution. My parents wanted for me what they did not have access to and emphasized the importance of pursuing a university education. Although I could not and still cannot afford university, I was fortunate enough to receive funding for my undergraduate and postgraduate studies, which is representative of both the increased access to opportunity in democratic South Africa, but also the existence of a ‘missing middle’. I have had the opportunity to travel to different parts of the world, primarily South East Asia, which has allowed me to engage with different people and be exposed to the impact of historical injustice. My background in debating at league, provincial, national and international competitions and attending conferences with diverse cultural and racial representation have allowed me to engage with the world critically and intentionally.

Cassia Holtz: I am a health and climate change researcher based in Johannesburg, South Africa. My positionality is shaped by being a highly educated white person from South Africa who holds both humanities and science-based degrees. My research is generally a mixed methods approach within both human and physical geography disciplines. I would call myself a global citizen with a family history across four continents and as a result have travelled extensively on all continents except Antarctica. My positionality is shaped by spanning Global South/North divides within my extended family and having personal histories in both. I would call myself a historical climatologist, using alternative resources to build up climate and health records in places in the world where official records have not been well-preserved.

Carmen Kganane: I am a straight Coloured woman who grew up in a Coloured community. A Coloured mother and a Black father. Both educated, with two degrees each. How they grew up shaped how I would grow up. In the midst of the Apartheid era, both my parents were able to obtain two degrees. It was a privilege for them to be granted such an opportunity. By being born after Apartheid, I knew university was somewhere I was going to go but I never imagined becoming the academic I am today.

Having obtained a Bachelor’s degree in Geography and Environmental Studies, an Honours in Environmental Studies and now working on my Masters in Geography, I understand what a privilege it is to be involved in academic spaces that may contribute to shaping my identity in the world of academia. A large portion of my positionality springs from being part of a racial group that is not well represented in the academic space.

Alexi M. Marinaki: I am a 26 year old straight Caucasian male was born in Johannesburg, South Africa where I currently live. I am of Greek nationality with family from Cyprus and Egypt as well. It is necessary to declare that the history of these three countries defines my social, political and economic views about the world. Many of these views both contrast and align with that of South African people. This forces me to constantly engage with the relationship between the various backgrounds of people in South Africa and my own. Many of my

family members from Greece, Egypt and Cyprus have experienced the complex socio-political arena that shapes our world. This is a facet of my own history that I actively engage with.

My education comprises a bachelor's degree in Geography and International Relations, an honours degree in Environmental Studies and a master's degree in Geography. Throughout my academic career thus far, I have shifted between qualitative and quantitative research where my interests were aligned. I feel it is valuable to engage with both sides of the research coin in an attempt to fully understand this complex, beautiful and, sometimes strange world we live in.

Chanice Mavudzi: I am a half Zimbabwean, half Dutch mixed-race woman, born and raised in South Africa. I hold a bachelor's degree in Environmental and Geographical Sciences and Sociology, with a focus on physical geography, atmospheric science, and African studies, as well as an honours degree in Geography. I am currently pursuing a Masters degree in Geography looking at thermal risk faced by construction workers in South Africa. As a Black woman in science, I recognize the importance of representation and inclusivity in research.

My positionality is deeply shaped by my identity as a mixed-race South African woman, which informs both my worldview and my approach to research. Growing up in a country marked by complex racial and social dynamics has made me especially aware of issues of equity, environmental justice, and community resilience.

Palesa Molefe: My perspective is greatly shaped by my background, education, and other experiences. I am a young Black woman from Springs, a small town east of Johannesburg, South Africa. While I have not directly experienced some of the socioeconomic challenges in my community, I am aware of issues such as poverty, youth unemployment, substance abuse, and environmental degradation that are prevalent around me. As an emerging academic with a science undergraduate degree in Environmental Studies and Applied Ecology, and a honours degree in Environmental Studies from the University of the Witwatersrand, South Africa, one of the top universities in the country. I am currently pursuing a Master of Science degree in Geography, specialising in health biometeorology. I believe I am well-equipped to understand the complexities faced by developing countries, while also envisioning incorporating an indigenous knowledge perspective into my studies. Although I have not yet had the opportunity to travel outside my country, I have explored several provinces within it, including the Free State, Limpopo, North West, Mpumalanga, and KwaZulu-Natal. Each of these regions has a rich history of colonialism, apartheid, and tribal influences that continue to impact the current socioeconomic climate.

Ogone Motlogeloa: I'm a heterosexual Black African woman from South Africa, shaped by a strong educational foundation. My parents were both schoolteachers who instilled in me and my older siblings a deep respect for learning and critical thinking. That upbringing laid the groundwork for my academic journey, which led to a PhD in Health Biometeorology. Although I now work in academia, I carry the grounding and humility of growing up in a teaching household, where learning was a shared value, not a career path. My partner is a data scientist, and our conversations often spark new ways of thinking, deepening my appreciation for evidence-based approaches and cross-disciplinary collaboration. Over the years, I've had the privilege to travel for work and research, which has expanded my perspective on how climate and health intersect in vastly different ways across communities and regions.

Nkosi Muse: I am a straight, cisgender American male, born to formerly disadvantaged Black parents who worked hard to provide a stable life for their children. As such, I have never considered myself underprivileged; however, as a Black man, I do belong to a historically marginalized community not only globally, but especially in the United States. As such, throughout my scientific career, including earning a PhD, I have frequently been one of the few, if not the only, scientists of color in the room. Trained in meteorology, I consider myself a climate and environmental scientist/policy expert, as well as a passionate advocate for environmental/climate justice and equity in STEM and science education.

Although I have never lived in the global south, I have worked with and to assist socially vulnerable communities in the United States who could be considered "global south communities," although they live in the global north. Because of this experience, I approach science and policy work that has social implications with culturally inclusive, culturally relevant, and culturally sensitive methods to ensure maximum positive outcomes and prevent exploitation of knowledge.

Amanda Ndiweni: My positionality is shaped by my identity as a black-female South African, born and raised in the city of Johannesburg and having moved between rural Taung in the Northwest province and a low-income community in Johannesburg South. I have been raised by parents who pursued their education during my high school years (the later stages of their own lives). I am educated with degrees in both science and humanities disciplines (BA Geography and Sociology & BSc Geography Honours) and have only travelled within the African Continent (Zimbabwe, Botswana).

My education and lived experience amongst low-income workers, rural communities, being surrounded by people largely impacted by the apartheid history and only being exposed to more affluent areas of South Africa in the later years of my life have shaped my perception of the global South and the value I place on historical context and the views held by the marginalised in the “Global South” regions.

Leonardo Brandão do Prado: As a Brazilian, gay man from the periphery of Rio de Janeiro, and someone who identifies politically with the left, who studied my entire life in fully free public schools and universities until my Masters within a Bachelor in Geography and a masters in Environmental Planning and Management, Geography at Federal University of Rio de Janeiro - UFRJ and today, as an international student pursuing my PhD at Arizona State University in the United States, working with street vendors and their exposure to the heat. I acknowledge that the traces of the colonial legacies persist in the history and the materiality of cities in the Global South.

Pippa Pryor: My positionality is shaped by being a white straight female born and raised in Sydney Australia. I am highly educated, with a Bachelor of Rural Science (First Class Honours) and am in pursuit of a PhD focussing on heat tolerance in cattle. My perspectives created during my upbringing in Sydney, were altered throughout my higher education due to moving to rural New South Wales for my bachelor’s degree and then to a remote town in Queensland for the first year of my PhD. I am now located in a small town of 3000 people, inland from Brisbane. I have also travelled to the USA, the UK, Belgium, France, the Caribbean and New Zealand as well as Vietnam, the Cook Islands, Kenya and South Africa. I have travelled to these locations for both personal and educational purposes (WWI scholarship).

Sarah Roffe: I am a straight, white South African female, and as of July 2025, I am 32 years old. I was born in Johannesburg and raised as a middle-class South African in the suburban area of Alberton, attending government schools before studying Geography and Archaeology as my BSc majors, alongside subjects like Physics, Chemistry and Statistics, at the University of the Witwatersrand. While my parents supported my undergraduate studies, I relied on funding support throughout my postgraduate (MSc through to PhD) and postdoctoral studies, which focused largely on the climate science discipline on quantitative analyses of rainfall seasonality, climate change and variability, palaeoclimate model output, extreme temperature events, and thermal comfort, all within the southern African context. As an early-career researcher, nearly six years post-PhD, I work as a senior researcher at the Agricultural Research Council, focusing on agrometeorology and agroclimatology to connect scientific understanding with the realities of farming communities in South Africa. I work closely with the South African Department of Agriculture, using my research, which is strongly data-oriented, to inform policy development and implementation. I have travelled internationally, to Global North and South countries, on numerous occasions, both for work and leisure, but my research and professional identity remain firmly rooted in South Africa. Having recently moved from suburban Alberton to the more rural farming area of Scheerpoort near Hartbeespoort, I recognise that this shift also shapes how I engage with the agricultural sector and the communities I serve through my research.

Mahdieh Saed: I am a Middle Eastern woman, born and raised in Iran. I hold a Master’s degree in Urban Planning from my home country, and I am currently pursuing a PhD in Geography at the University of Waterloo, with a focus on extreme heat and public health.

My perception of the Global South has been shaped by my lived experience as a first-generation university student, raised in a middle-class family, impacted by the unstable economic and political conditions in my country, and witnessed significantly increasing inequities in my surroundings. In addition, migrating to a developed country (Canada) to pursue my PhD and being exposed to diverse cultures and perspectives has further shaped my positionality.

Raghid Shehayeb: I am a 32-year-old male from the town of Aley, Lebanon. I come from a middle-class family and lived and studied in the country until 2018. I have a Bachelor's degree in civil and environmental engineering. After completing my bachelors, I struggled to find a job in the field I was interested in and hence, worked as a coordinator in a local NGO on peace building after being a volunteer there for several years. This shifted my perspective from a fully engineering standpoint to more societal relevant topics and drove me to search for interdisciplinary master’s programs related to water, sustainability and the environment.

In 2018, I was admitted to a Master’s degree in Integrated Water Resources Management in Cologne, Germany. The focus of the studies was on the MENA region, and I did an exchange semester in Jordan for four years under this program. The case study of my master thesis was in Beirut, Lebanon, on urban water security. Thereafter, I received a scholarship for a Doctoral degree in TU Dresden and the Leibniz institute of Ecological Urban and Regional Development, in Germany. The ongoing doctoral research is on drought and heat multi-risks for ecosystem services of urban green infrastructure. Most recently, I took on a position as a scientific coordinator of the Leibniz Research Network “Earth & Societies”.

The six years in Germany increased my understanding of the global south and north disparities, and the different challenges faced by global regions. Additionally, travelling during the studies to countries such as Turkey, Qatar, Jordan, France, Greece, Albania, Spain broadened my perspective on these countries. The most recent trip to South Africa showed me many commonalities between global south countries, but also certain specificities that were unknown to me.

Adriaan J. van der Walt: As a researcher situated within the academic field of geography in South Africa, my positionality is shaped by my social, cultural, and institutional context. I am a white, Afrikaans-speaking South African academic working at the University of the Free State in Bloemfontein, an institution with a complex history and a clear contemporary commitment to transformation and social justice. My academic background and disciplinary training inform my analytical approach, while my lived experiences in a post-apartheid society heighten my awareness of structural inequalities and the legacies of colonialism and segregation.

Mukhtaar Waja: I am a 26-year-old heterosexual, South African male, of Indian ethnicity. I have lived in South Africa my entire life, having been raised in an upper middle-class family. I am currently a PhD Candidate at the University of the Witwatersrand, conducting research within the field of health biometeorology. I am areligious, and my politics are left-leaning. I have engaged in volunteer work with charitable organisations in Johannesburg, and through this received exposure to the inequity and impoverishment present in the city. I also witness such inequity daily, during my regular and varied commutes throughout the Gauteng province. I have also travelled throughout South Africa, and am familiar with many of the major cities throughout the country. My knowledge of countries outside of South Africa is largely informed by my consumption of news media – Al-Jazeera being an oft-used news source – and my international travels, all of which have been of a personal and not professional nature. I have holidayed in both the megacities and small villages of India; I have stayed in the city centre of Cairo, Egypt; I have visited Istanbul, Turkey; and I have holidayed in Bangkok and Phuket, Thailand.

Michael J. Allen, Ph.D.: As a Geographer, academic professional, and curious citizen of planet Earth, my perception of the world is shaped by my background and life experiences which inform how I engage and interpret the world. A white man from the United States, a person who has traveled extensively, and one who now lives in Baltimore, I acknowledge the privileges and limitations my worldview brings, and I strive to remain critically reflective and responsive to diverse experiences. My work is grounded in a commitment to amplifying the often-unheard voices in the face of environmental change. One's worldview is based on personal experience, observation, and lessons learned. Visiting a place today is not the same as tomorrow, yet our understanding of a place is predicated on these geographic imaginations. Additionally, one city is not representative of a larger place. Such interactions are solely my non-perfect, non-complete "reality" of the world.

Jennifer Fitchett: I am a white female full professor of geography, living and working in South Africa. I have lived in South Africa my whole life, born and raised in Johannesburg where I currently reside and work. I studied in Cape Town for my three year undergraduate, and spent four months of my PhD in London. I have, however, through work and vacation had the privilege of visiting many countries, including Thailand, Turkey, Austria, Lesotho, Namibia, Mozambique, the United States of America, Brazil, Egypt, Ireland, Mauritius and Reunion. I work in a discipline that engages critically with the expansion of research into the Global South and led by the Global South, across physical and human sciences, a field in which I have published. My academic research spans Southern Africa and the Middle East.

Peter Crank: I am a white male from North America, specifically the U.S., who is also highly educated, even by U.S. standards where only about 1% of the population has a Ph.D. My educational background is in the quantitative physical geography and atmospheric sciences. However, during my PhD I undertook more "medical" research and developed an appreciation for qualitative methods of research. I am thus still on a journey of unlearning the ideology that quantitative approaches are "more scientific" than the qualitative. Additionally, I have had the privilege to have lived in multiple states within the US and I have visited nearly every state in the U.S and have lived in other countries as well, including Singapore and have traveled throughout Southeast Asia, Europe, and North America. This has transformed my perspective on how things "should" and can be done (societally, scientifically, etc.). A transitioning piece of my positionality is that for me, it wasn't until I was in my PhD that I met my wife, who is an African American descended from slaves. My relationship to and with her greatly shifted my perspectives on society. While there was an awareness before of racism within the US and took the time myself, even before I met her, to visit civil rights museums and to learn about the racialized history of the US, being married to somebody whose identity is racialized in such a brutal capacity historically changes the way that I enter into these spaces and the way that I think about geographies of disparity and inequity.
